# A Wild Allele of *Pyrroline-5-Carboxylate Synthase1* Leads to Proline Accumulation in Spikes and Leaves of Barley Contributing to Improved Performance Under Reduced Water Availability

**DOI:** 10.3389/fpls.2021.633448

**Published:** 2021-02-25

**Authors:** Felix Frimpong, Carel W. Windt, Dagmar van Dusschoten, Ali A. Naz, Michael Frei, Fabio Fiorani

**Affiliations:** ^1^Institute of Bio- and Geosciences, IBG-2: Plant Sciences, Forschungszentrum Jülich GmbH, Jülich, Germany; ^2^CSIR-Crops Research Institute, Kumasi, Ghana; ^3^Department of Plant Breeding, Institute of Crop Science and Resource Conservation, University of Bonn, Bonn, Germany; ^4^Institute of Agronomy and Plant Breeding, Justus-Liebig-Universität Gießen, Gießen, Germany

**Keywords:** barley, introgression lines, seed yield, proline accumulation, *pyrroline-5-carboxylate synthase1*, water stress

## Abstract

Water stress (WS) during spike development strongly affects final grain yield and grain quality in cereals. Proline, an osmoprotectant amino-acid, may contribute to alleviating the effects of cell and tissue dehydration. We studied five spring barley genotypes contrasting in their drought response, including two introgression lines, *S42IL-143* and *S42IL-141*, harboring a *Pyrroline-5-carboxylate synthase1- P5cs1* allele originating from the wild barley accession *ISR42-8*. We tested the hypothesis that barley genotypes harboring a wild allele at *P5cs1* locus are comparatively more drought-tolerant at the reproductive stage by inducing proline accumulation in their immature spikes. At the booting stage, we subjected plants to well-watered and WS treatments until physiological maturity. Several morpho-physiological traits had significant genotype by treatment interaction and reduction under WS. Varying levels of genotypic proline accumulation and differences in WS tolerance were observed. Spike proline accumulation was higher than leaf proline accumulation for all genotypes under WS. Also, introgression lines carrying a wild allele at *P5cs1* locus had a markedly higher spike and leaf proline content compared with the other genotypes. These introgression lines showed milder drought symptoms compared with elite genotypes, remained photosynthetically active under WS, and maintained their intrinsic water use efficiency. These combined responses contributed to the achievement of higher final seed productivity. Magnetic resonance imaging (MRI) of whole spikes at the soft dough stage showed an increase in seed abortion among the elite genotypes compared with the introgression lines 15 days after WS treatment. Our results suggest that proline accumulation at the reproductive stage contributes to the maintenance of grain formation under water shortage.

## Introduction

The ability of crops to withstand WS is a critical aspect of the potential impact of climate change on crop productivity in agricultural systems (Ferguson, 2019; Gupta et al., 2020). Plants use different strategies to cope with water shortage: avoidance, escape, or tolerance. The escape strategy is an adaptive mechanism that involves rapid plant development to enable the completion of the full life-cycle before a drought event can occur (Shavrukov et al., 2017). In cereals, drought escape is associated with a short vegetative stage and early flowering time. The avoidance strategy involves minimization of water loss and optimization of water uptake, which comprises physiological responses that improve photosynthetic water use efficiency, such as stomatal closure (Blum, 2005; Basu et al., 2016; Fahad et al., 2017; Rodrigues et al., 2019), stay green (Tardieu et al., 2018; Wasaya et al., 2018; Sallam et al., 2019), deeper rooting (Arai-Sanoh et al., 2014; Lynch and Wojciechowski, 2015; Kebede et al., 2019), or the accumulation of osmolytes and osmoprotectants (Bandurska et al., 2017).

Drought is known to profoundly affect plant metabolism (Templer et al., 2017). The accumulation of compatible solutes such as sugars, proline, fructans, glycine betaine, and polyamines is associated with increased drought tolerance in plants (Bhaskara et al., 2015; Templer et al., 2017; Trovato et al., 2019). Drought stress increased proline concentration about 10-fold in the leaves of monocotyledons such as rice (*Oryza sativa*) and dicotyledons species such as *Brassica oleracea* seedlings (Dien et al., 2019; Podda et al., 2019).

Proline is synthesized from glutamate by the action of three enzyme coding genes, pyrroline-5 carboxylate synthase (*P5CS*), pyrroline-5-carboxylate synthase-2 (*P5CS2*), and pyrroline-5-carboxylate reductase (*P5CR*) (Bhaskara et al., 2015; Trovato et al., 2019). Several reports investigated the proline biosynthetic pathway and the corresponding key enzymes *P5CS* and *P5CR* have been well characterized (Forlani et al., 2015; Choudhury et al., 2017; Abdel-Ghani et al., 2019; Kamal et al., 2019). In higher plants, the most rate-limiting enzyme for proline synthesis is pyrroline-5-carboxylate synthase (Trovato et al., 2019). Proline biosynthesis occurs under both non-limiting and limiting growth conditions (Cattivelli et al., 2011; Nieves-Cordones et al., 2019). Under non-limiting growth conditions, proline is used in protein biosynthesis to maintain the housekeeping function of the cell (Hoffmann et al., 2017). Proline accumulation under WS is accompanied by the increased expression of *P5cs1* (Muzammil et al., 2018). The expression of both *P5cs1* and *P5CR* is increased in leaves when barley is exposed to drought, resulting in enhanced proline synthesis in the chloroplast, whereas *P5CS2* is primarily linked to proline synthesis in the cytosol (Sayed et al., 2012).

In barley, introgression lines carrying naturally occurring alleles (cross between *Scarlett* and wild type *ISR42-8*) associated with proline accumulation and leaf wilting under drought stress conditions were reported previously (Sayed et al., 2012; Honsdorf et al., 2014, 2017; Naz et al., 2014). To test the hypothesis that the allelic variant of *P5cs1* controls the drought-inducible QTL (*QPro.S42-1H*) in the donor parental line and progenies, Muzammil et al. (2018) performed a series of phenotypic evaluations. They demonstrated that the progeny introgression lines maintained leaf water content and photosynthetic activity longer compared with those of the cultivated parents under drought conditions. Nonetheless, to understand the integrative networks of plant metabolites and signaling molecules, the sites of their biosynthesis and action must be clarified (Kuromori et al., 2018). Understanding the specific target sites regulating seed filling events in leaves and seeds and how they are affected by abiotic stresses is imperative to enhance seed quality (Sehgal et al., 2018). Knowledge of the physiological, biochemical, and genetic mechanisms which govern seed filling under stressful environments helps to devise strategies to improve stress tolerance (Sehgal et al., 2018; Abdelrahman et al., 2020). Little attention has been paid to the role of proline in the reproductive organs (Heuer, 2016), especially spikes or seeds, and the changes in its concentration in different plant organs under WS. In this study, we addressed this knowledge gap by measuring spike and leaf proline content, changes in photosynthetic performance, and assessing barley seed abortion and GF under WS using MRI at the reproductive stages.

We tested the hypothesis that drought-induced proline accumulation in spikes of barley genotypes harboring the wild variant of *P5cs1* improves drought tolerance as measured by seed number and final yield in greenhouse experiments. To this end, we characterized a panel of contrasting elite genotypes and *P5cs1*-introgression lines and monitored morpho-physiological responses after water withdrawal during reproductive development.

## Materials and Methods

### Plant Material

Four two-row and one six-row spring barley genotypes *S42IL-141*, *S42IL-143*, *Scarlett*, *Barke*, and *HOR10151* were chosen for this study based on the initial screening, their genetic background, breeding history, agronomical importance, and previously reported yield under drought stress conditions. *S42IL-141*, *S42IL-143* carried chromosomal introgressions at *P5cs1* locus from wild barley accession *ISR42-8* (Muzammil et al., 2018). *Barke* and Scarlett are elite German cultivars. *HOR10151* is a six-row traditional landrace known to escape drought when grown at high elevations of the Libyan region where it originated.

### Growth Conditions and Water Stress Treatment

Two experiments were conducted in a greenhouse (Forschungszentrum Jülich, Germany, IBG2: Plant Sciences; 50°55′17.36″N, 6°21′45.61″E) in two consecutive years, June – October of 2018 and 2019 under long-day conditions (16 h/8 h day/night). Extra illumination (SON-T AGRO 400, Philips, Amsterdam, Netherlands) was automatically supplied when the ambient light intensity inside the greenhouse was <400 μmol m^–2^ s^–1^, between 06:00 and 22:00 h. The average minimum and maximum greenhouse daily light integral (DLI, mol m^–2^ day^–1^) were 6 and 13 in 2018 and 3 and 12 in 2019 (Supplementary Figure 1). Day/night minimum and maximum temperature of the greenhouse was ∼ 20 ± 4°C and 30 ± 4°C during the day and ∼ 16 ± 2°C and 20 ± 2°C during the night, respectively. Cumulative growing degree days were calculated assuming a base temperature of 0°C (Miller et al., 2001; Hecht et al., 2019) from the time of emergence until the ripening stage. These were 2,155 and 2,059 degree days in 2018 and 2019, respectively. Pre-germinated seeds of five genotypes were transplanted into 1.5 L pots upon reaching the three leaves stage. Peat soil (Einheitserde, “null type”) was used for both experiments arranged in a 5 × 2 factorial randomized complete block design with six and fifteen replicates per genotype and treatment in 2018 and 2019, respectively. Three tablets of the 5 g Osmocote Exact slow-release fertilizer (14-8-11; N – P_2_O_5_ – K_2_O + 2 MgO + trace elements) were applied per plant in three aliquots starting 2 weeks after transplanting. Pests and diseases were controlled chemically according to established greenhouse practices.

Water was administered with the help of an automated drip irrigation setup at the greenhouse (Netafilm, Adelaide, SA, Australia), watering the pots twice daily. Starting from the booting stage (BBCH-scale 41, Meier, 2001), all genotypes were subjected to two irrigation regimes. The treatments were WW and WS. We applied WS by first withholding water for the selected plants for 48 h, and then adjusted the irrigation volume three times per week to maintain target soil moisture per treatment. WW plants were irrigated daily (400 mL per plant) approximately to 50% g/g gravimetric soil water content in two aliquots per day; WS plants were irrigated daily (120 mL per plant) approximately to 20% g/g gravimetric soil water content in two aliquots per day (Supplementary Figure 2). Soil water content was monitored with the aid of a three-pin time-domain-reflectometry soil moisture Theta ML2 probe (Delta-T Devices Ltd., United Kingdom), after calibration (*R*^2^ = 0.94) from volumetric to gravimetric soil water content. The corresponding soil water potential (Ψ soil) values of WW and WS were −0.001 and −1.5 MPa, respectively. These soil water potential values were estimated using eight-point water retention curves that were fitted with van Genuchten model (van Genuchten, 1980).

### Morphometric and Physiological Measurements

Twice a week, two plants of each genotype were dissected under a stereomicroscope to observe spike developmental stages and characterize treatment effects. The stereomicroscope (Leica MZ12 stereo microscope, Germany) was equipped with a 1.0 × planochromatic objective and with 10 × eyepieces, a numerical aperture of 0.125, and a resolution of 375 line pairs/mm. The number of days to reach each stage of development was counted for both WW, and WS treated plants. Plant height and tiller number were determined at harvest. At harvest (20 days after WS) yield traits such as spike number, spike length (cm), spike weight (g), total grain weight (g), grain number, shoot fresh weight (g), and shoot dry weight (g) were determined on a per plant basis. A DSI for dry grain yield (g) per plant was calculated using the following formula (Haddadin, 2015):

$$D\,S\,I=\frac{1-\frac{Y\,D}{Y\,P}}{1-\frac{\text{WD}}{\text{WP}}}$$

where:

YD, mean yield of individual genotype under the WS condition.

YP, mean yield of individual genotype under the WW condition.

WD, mean of all genotypes under the WS condition.

WP, mean of all genotypes under the WW condition.

At harvest, fresh, and dry weight per plant (g) were determined for shoot and root (after washing) biomass. Percentage relative leaf water content of fully expanded leaves was calculated:

$\%relativeleafwatercontent\left(RWC\right)=\frac{\left(\mathrm{fresh}\,\mathrm{leaf}\,\mathrm{weight}-\mathrm{dry}\,\mathrm{leaf}\,\mathrm{weight}\right)}{\left(\mathrm{turgid}\,\mathrm{leaf}\,\mathrm{weight}-\mathrm{dry}\,\mathrm{leaf}\,\mathrm{weight}\right)}\times 100$, according to Barrs and Weatherley (1962), fifteen days after the WS application.

WS treated leaves were scored for wilting one time forenoon, fifteen days after the onset of treatment using a scale from 0 to 9. A score of 0 indicated no wilting and 9 is fully wilted (De Datta et al., 1988; Sallam et al., 2019). Gas exchange parameters (net CO_2_ assimilation – *A*, μmol CO_2_ m^–2^ s^–1^, stomatal conductance – *gsw*, mmol H_2_O m^–2^ s^–1^, transpiration rate – *E*, mol H_2_O m^–2^ s^–1^, and intrinsic water use efficiency (*A/gsw)* –iWUE, μmol CO_2_ mmol^–1^ H_2_O) were measured on the youngest leaf directly below the flag leaf on the main stem at one-time point during the experiment of 2018 (15 days after WS). The flag leaf of the main stem was used for the gas exchange measurements in 2019 at 3, 9, and 15 days after WS application. Fifteen and six plants per genotype per treatment in 2019 and 2018, respectively, were used for the gas exchange measurements. Leaves were clamped in the MultiPhase Flash ^TM^ fluorometer chamber (551065), 10% blue light, 6 cm^2^ LiCOR cuvette, and exposed to PPFD of 1,500 μmol m^–2^s^–1^, Airflow (500 mmol s^–1^), block temperature of 25°C, 400 ppm of CO_2_, humidity (RH) ranging between 50 and 65% using a LiCor 6,800 (LiCOR Inc., Lincoln, NE, United States). Instantaneous photosynthesis and GSW were measured after steady-state gas exchange conditions inside the cuvette were reached. Measurements were completed between 10:00 am and 3:00 pm during the day for all barley plants by following the randomization order of the experimental layout to account for the possible effects of time of day on the measurements, which could spuriously bias genotypic values and variability estimation as well.

### Magnetic Resonance Imaging

The magnetic resonance imaging scans were carried out using a custom-built, vertical bore 4.7 T MRI scanner, driven by a Varian console VNMRS, vertical wide-bore MRI system (Varian Inc)^1^. The system was equipped with a quadrature to transmit/receive coil with an inner diameter of 100 mm and a 300 mT/m gradient system. The main spikes at the dough stage (BBCH-scale, 83) were collected together with a section of the stalk (>20 mm). The cut spikes were placed in a vial with tap water directly after excision. A robotic system (MiniLiner 3.0, Geiger Handling GmbH and Co. Kg, Jülich, Germany) was used to carefully lower and center the specimen into the MRI scanner. 2D images of developing spikes were acquired with an in-plane spatial resolution of 0.3750 and 0.1875 mm, using multi-spin-echo sequence based on the following set routines; 32 echoes, 1.5 s repetition time, echo time of *n* × 8 ms, two averages, 512 × 256 image matrix, a field of view of 100 (read direction) by 50 mm (phase direction), at a slice thickness of 50 mm. The acquired datasets show amplitude images of water content per pixel (Edzes et al., 1998). Each spike was imaged for 12 min. An additional 10 min of preparation and setting the sample to the center of the magnet were required. For all spikes, the MRI images presented are amplitude parameter maps of a single echo image in gray values in their sagittal orientation after analyzing using image reconstruction set scripts from Spyder, scientific programming in Python 3.6.

### Proline Determination in Barley Leaves and Spikes

Proline concentrations were determined based on the protocol of Bates et al. (1973), with few modifications. Six replicates of each of the flag leaf and immature spike samples were collected from both treatments and genotypes and immediately submerged in liquid nitrogen. Samples were stored at −80 °C. Samples were pulverized using pestle and mortar on ice. One hundred mg of the pulverized samples were weighed and extracted with 1.5 ml of 3% salicylic acid in chilled 2 mL tubes, vortexed, and centrifuged at 12,000 rpm for 10 min. Five hundred μL of the supernatant was directly transferred into cylindrical glass tubes (fitted with lids) on ice and 500 μL of glacial acetic acid and 2.5% ninhydrin reagent added. The mixture was then vigorously vortexed and incubated for 1 h in a water bath at 95 °C. The reaction was quickly terminated on ice. 1.5 mL of toluene was added, and the mixture was kept at room temperature for 30 min after vortexing. One hundred μL of the upper phase was then pipetted into 96 well plates, and the absorbance at 520 nm measured using a microplate reader (Synergy^TM^ 2 Multi-Mode, BioTek, Winooski, Vermont, United States). An empirical calibration curve based on eight points of proline standard concentrations (0, 10, 20, 30, 50, 70, 90, and 100 μg/g) yielded a linear regression between proline concentration and the measured absorbance at 520 nm (*R*^2^ = 0.998). This linear model was subsequently used for proline concentration calculation in the samples.

### Statistical Analysis

All data were subjected to normality (Shapiro Wilk test) and variance homogeneity tests (Levene’s test). Power transformation (Box and Cox, 1964) was performed for the gas exchange and proline measurements because normality or homogeneity conditions were not met. The main effects of genotypes and WS treatments, along with their corresponding interactions, were tested first using a three-way analysis of variance. We found treatment × genotype × year interaction effect for all data (except for spike length, Supplementary Data Sheet 2). Therefore, we proceeded to analyze the data using a year-specific two-way analysis of variance. We used the generalized linear model;

μ_*ijk*_ = μ + α_*i*_ + β_*j*_ + (αβ_*ij*_) + ε_*ijk*_, where;

μ, grand mean.

α_*i*_*and*β_*j*_, main effects of WS treatment and genotypes of the *ith* and (αβ_*ij*_) levels.

*jth*, interaction effect.

ε_*ijk*_, error term.

built-in the “Agricolae” package of “R” statistical software, version 3.6.1 (R Core Team, 2019). Tukey’s HSD (Honest Significant Difference test) was used to determine significant differences between treatment and genotypic means within plant traits. Spearman correlation coefficients for pair-wise comparisons for selected traits were computed.

## Results

### Effects of Water Stress on Barley Morphology and Physiology

**FIGURE 1:**
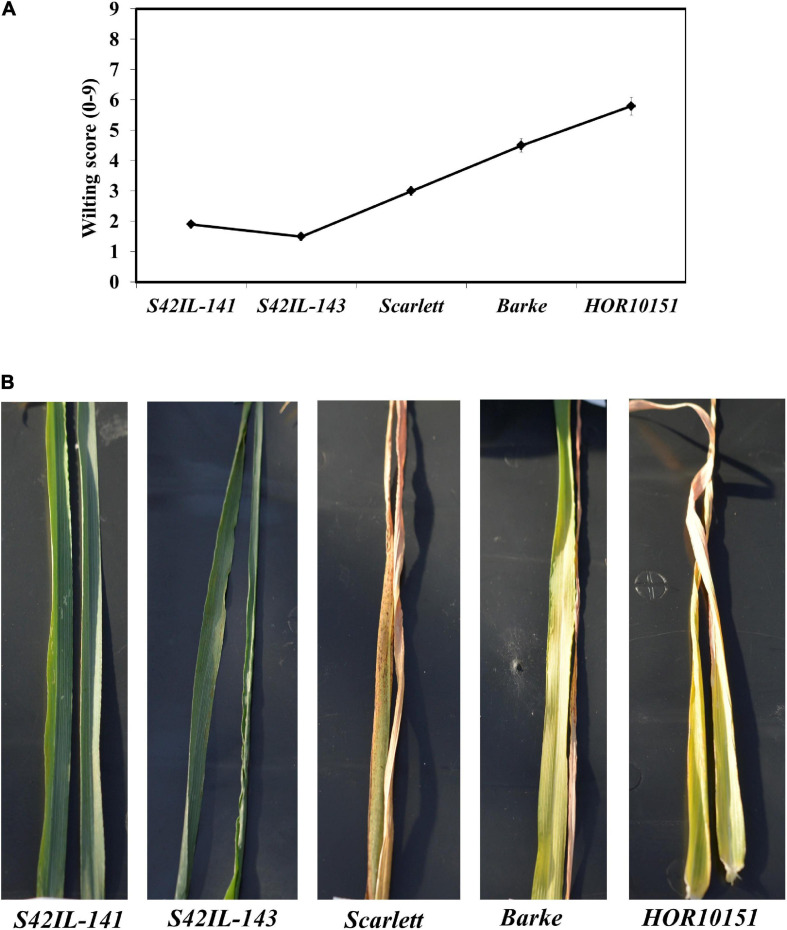
Wilting severity by the different genotypes recorded forenoon, 15 days after onset of water stress adapted from De Datta et al., 1988. **(A)** Is a line graph depicting wilting scores for each genotype. **(B)** An illustration of the wilting score/drought symptoms as shown by representative leaves (leaf 5 and 6 fully expanded) of the different genotypes.

Pronounced leaf wilting was observed under WS for all the genotypes and treatments (Figure 1). However, the two introgression lines *S42IL-143* and *S42IL-141* showed milder wilting symptoms (−40%) than the elite barley types *Barke, Scarlett*, and *HOR10151* (Tables 1, 2). The six-row barley type, *HOR10151* showed higher susceptibility to wilting than the introgression lines with more than 50% of its leaves drying 15 days after stress application (Table 2 and Figure 1). Averagely, WS *S42IL-143* and *S42IL-141* had a wilting score of 2 or less while the elite lines were 3 and above (Table 2). None of the WW plants showed any wilting symptoms (Table 1). Results from both 2018 and 2019 experiments showed that introgression lines *S42IL-143* and *S42IL-141* maintained their RWC (>70%) both under WS and WW conditions Table 2. Differently from the WW conditions, elite cultivars showed smaller variations in RWC under WS (Tables 1, 2). In 2019, *Barke* and *HOR10151* had the lowest RWC (∼35%, Tables 1, 2) under WS.

**TABLE 1:** Two-way analysis of variance of the plant traits in 2018 and 2019 under control, drought, genotypes, and genotype × treatment interaction, at the end of water stress application.

Traits	2018					2019				
	Control	Drought	Treatment	Genotype	G × T	Control	Drought	Treatment	Genotype	G × T
Plant height (cm)	92.9	75.9	***	NS	NS	93.3	67.8	***	***	NS
Tiller number	17.50	13.97	***	NS	NS	21.40	11.39	***	*	NS
Wilting/drought score	0.00	2.85	***	***	NA	0.07	3.36	***	***	NA
Relative leaf water content (%)	87.5	74.4	***	***	***	85.9	55.7	***	***	***
Spike number	18.35	10.00	***	***	*	26.40	16.39	***	***	***
Grain weight/plant (g)	12.57	2.91	***	***	***	14.22	4.37	***	***	***
Grain number per the main tiller	25.27	18	***	***	NS	28.84	11.05	***	***	***
Length of the main spike (cm)	10.61	8.68	***	***	NS	10.01	7.82	***	***	***
Shoot fresh weight (g)	59.01	46.13	***	***	**	35.8	19.9	***	***	NS
Shoot dry weight (g)	27.63	23.56	***	***	**	18.22	12.15	***	***	*
Root dry weight (g)	4.99	3.91	***	***	NS	3.99	2.91	***	***	NS
Root/shoot ratio (g)	0.18	0.17	NS	NS	NS	0.22	0.24	NS	NS	NS
A (μmol m^–2^ s^–1^)	28.83	13.13	***	***	***	21.51	6.08	***	***	***
E (mol m^–2^ s^–1^)	9.9E^–3^	3.6E^–3^	***	***	***	9E^–3^	2E^–3^	***	***	***
Ci (μmol mol^–1^)	260.86	176.54	***	***	***	239	216	*	NS	NS
VPDleaf	2.34	3.19	***	***	***	2.22	2.32	***	NS	NS
gsw (mol m^–2^ s^–1^)	0.43	0.11	***	***	***	0.28	0.063	***	***	***
iWUE (μmol CO_2_ mmol^–1^ H_2_O)	68	118	***	NS	*	81	95	***	***	***
ETR (μmol m^–2^ s^–1^)	163	99	***	***	***	139	100	***	***	***
Leaf proline (μg/g FW)	63	325	***	***	***	43	299	***	***	***
Spike proline (μg/g FW)	–	–	–	–	–	120	580	***	***	***

“*, **, and ***” – Follows the standard probability values (*P* ≤ 0.05, *P* ≤ 0.01, or *P* ≤ 0.001). Means of A, E, Ci, VPDleaf, gsw, iWUE, ETR, and proline were back-transformed to original values after transformation. A, net CO_2_ assimilation; E, transpiration; gsw, stomatal conductance; VPDleaf, vapor pressure deficit of the leaf; iWUE, intrinsic water use; efficiency, A/gsw; ETR, electron transport rate; FW, fresh weight; NA, not analyzed; NS, not significant.

**TABLE 2:** Differential biochemical and yield traits in response to water stress among the genotypes and drought treatments.

Year	Genotype	Well-watered				Water stress				
		% Relative leaf water content	Grain weight/plant (g)	Grain number per main tiller	Length of the main spike (cm)	% Relative leaf water content	Wilting score	Grain weight/plant (g)	Grain number per main tiller	Length of the main spike (cm)
2018	*Barke*	89.4 ± 1.07ab	13.7 ± 0.33ab	20.5 ± 1.77a	10.6 ± 0.11bc	56.5 ± 1.07c	3.3 ± 0.18b	1.4 ± 0.33f	6.3 ± 1.77b	8.3 ± 0.11d
	*HOR10151*	86.4 ± 0.88ab	9.8 ± 1.39c	27.33 ± 3.50a	6.2 ± 0.26e	57.2 ± 0.88c	4.9 ± 0.14a	2.8 ± 1.39ef	17.33 ± 3.50ab	4.9 ± 0.26e
	*Scarlett*	90.9 ± 1.64a	14.0 ± 0.90a	25.7 ± 2.02a	11.7 ± 2.06ab	86.1 ± 1.64ab	2.9 ± 0.15bc	2.3 ± 0.90ef	18.2 ± 2.02a	9.2 ± 2.06cd
	*IL141*	87.4 ± 0.69ab	11.3 ± 0.72bc	26.5 ± 1.65a	12.7 ± 1.65a	85.3 ± 0.69ab	1.9 ± 0.15cd	5.3 ± 0.72de	23.7 ± 1.65a	10.7 ± 1.65bc
	*IL143*	85.6 ± 0.95ab	14 ± 0.52a	26.3 ± 1.41a	11.8 ± 1.40ab	84.6 ± 0.95b	1.2 ± 0.16de	6.7 ± 2.54d	24.2 ± 0.52a	10.4 ± 1.40bc
2019	*Barke*	87.5 ± 2.04a	14.0 ± 0.38a	23.6 ± 0.75b	10.0 ± 0.21bc	27.1 ± 1.99d	4.5 ± 0.11b	0.5 ± 0.24e	2.3 ± 0.63e	7.27 ± 0.29e
	*HOR10151*	84.6 ± 1.77a	10.6 ± 0.36b	43.2 ± 1.85a	6.5 ± 0.19e	28.6 ± 1.85d	5.8 ± 0.15a	1.1 ± 0.20de	4.0 ± 0.70de	4.5 ± 0.16f
	*Scarlett*	82.4 ± 1.42a	13.5 ± 0.51a	27.1 ± 1.05b	11.1 ± 0.22ab	56.9 ± 1.22c	3.0 ± 0.12c	2.7 ± 0.43d	9.5 ± 1.66d	8.47 ± 0.20d
	*IL141*	84.2 ± 1.19a	13.6 ± 0.58a	24.4 ± 1.22b	11.1 ± 0.32ab	73.6 ± 0.73b	1.9 ± 0.10d	4.9 ± 0.30c	22.1 ± 1.32bc	10.1 ± 0.26bc
	*IL143*	86.2 ± 2.11a	13.3 ± 0.38a	25.9 ± 1.12b	11.27 ± 0.23a	84.7 ± 1.01a	1.5 ± 0.12d	6.7 ± 0.24c	17.3 ± 1.23c	9.23 ± 0.23cd

*Different letters indicate significant differences among treatments and genotypes based on Tukey’s HSD test (*P* ≤ 0.05) within a trait. Values are least-square means ± standard errors of six replicates for 2018 and 15 replicates for 2019. Control plants showed no wilting and therefore scored zero and were not analyzed.*

The spike developmental stages from booting, heading, and anthesis up to the onset of GF were delayed by at least one day under WS treatment for all genotypes (Supplementary Figure 3). Barley genotype *HOR10151* had the most considerable delay (three days difference between WS and WW plants, Supplementary Figure 3). Plant performance for all genotypes was significantly reduced for both experimental years (Table 1 and Supplementary Table 2). Relative to WW conditions, we observed a percentage reduction (%) of average plant height (18, 27), tiller number (19, 47), spike number (45, 38), grain number (30, 58), spike length (18, 22), grain weight (76, 76), RWC (15, 35), net CO_2_ assimilation (56, 72), GSW (74, 77), transpiration rate (63,76), and ETR (31, 28) (Supplementary Table 2) in 2018 and 2019, respectively.

**FIGURE 2:**
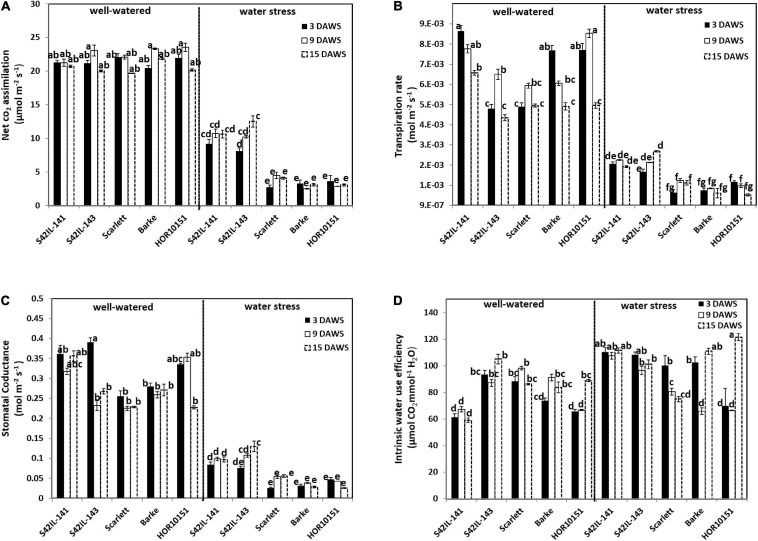
Gas exchange measurements of the different barley genotypes under well-watered and water stress treatment. Measurements were taken at 3, 9, and 15 days after water stress (DAWS), at booting, heading, and the onset of grain filling stages of spike development, respectively. Means and standard error bars are shown. The different letters indicate significant differences in treatment means based on Tukey’s (HSD) test (*n* = 15). **(A)** The net CO_2_ assimilation. **(B)** Transpiration rate. **(C)** Stomatal conductance. **(D)** Intrinsic water use efficiency.

Prolonged WS of fifteen days led to several leaves drying and reduced net CO_2_ assimilation by at least 50% (Figure 2A and Figure 1). Net CO_2_ assimilation, GSW, transpiration rate, and ETR were significantly reduced due to WS (Table 1). Significant genotypic variations were observed in the gas exchange parameters under WW conditions (Figures 2A–D). For instance, net CO_2_ assimilation in fully turgid leaves was between 22 and 24 μmol m^–2^ s^–1^ throughout the experiment period (Figure 2A). *Scarlett* had the lowest and *HOR10151* the highest net CO_2_ assimilation under WW (Figure 2A). On the other hand, the WS plants had a net CO_2_ assimilation rate between 2.5 and 10.7 μmol m^–2^ s^–1^ throughout the stress period (Figure 2A). *Barke* had the lowest value for net CO_2_ assimilation (2.5 μmol m^–2^ s^–1^), while the highest net CO_2_ assimilation rate was by *S42IL-141* and *S42IL-143* (10.7 and 12.5 μmol m^–2^ s^–1^) under WS, respectively (Figure 2A).

Transpiration rate was between 0.43 × 10^–2^ and 0.66 × 10^–2^ mol m^–2^s^–1^ under WW conditions, compared with 0.52 × 10^–3^ and 0.27 × 10^–2^ mol m^–2^s^–1^ under WS throughout the stress period (Figure 2B). *HOR10151* had the lowest transpiration rate, 0.52 × 10^–3^ mol m^–2^s^–1^ while *S42IL-143* transpired the most, 0.27 × 10^–2^ mol m^–2^s^–1^, 15 days after WS (Figure 2B). Stomatal conductance of fully turgid leaves was between 0.2 and 0.4 mol m^–2^ s^–1^ compared with WS leaves of 0.03 and 0.1 mol m^–2^ s^–1^ from booting to the onset of GF stages (Figure 2C). Introgression lines *S42IL-143* and *S42IL-141* maintained their photosynthetic activities by not only photosynthesizing at a higher rate several days after imposing WS but were also able to keep transpiring with low to moderate stomatal opening, ranging from 0.130 and 0.097 mol m^–2^ s^–1^ when the grain started filling (Figure 2C). These values were higher than those measured for *Barke, Scarlett*, and *HOR10151*, which were between 0.025 – 0.055 mol m^–2^ s^–1^ under WS (Figure 2C). Under WW conditions, intrinsic water use efficiency (iWUE) ranged between 59 and 105 μmol^–1^ CO_2_ H_2_O^–1^ (Figure 2D). iWUE of fully turgid flag leaves of *S42IL-141* was the lowest while *S42IL-143* was the highest across developmental stages. iWUE of the flag leaves of WS plants ranged between 65 and 122 μmol^–1^CO_2_ H_2_O^–1^. On average *Barke and Scarlett* had the lowest values at heading and onset of GF, respectively. *S42IL-141*had the highest iWUE under WS at booting and heading (Figure 2D). Interestingly, the iWUE of WS leaves of *S42IL-141* and *S42IL-143* increased by more than 20% compared with *Barke, Scarlett*, and *HOR10151* relative to their WW conditions (Figure 2D). iWUE of *Barke*, *Scarlet*, and *HOR10151* also increased marginally by 7% under WS on average. On average, elite genotypes *Barke, Scarlett*, and *HOR10151* had a lower increment of iWUE (7%) under WS than under WW conditions (Figure 2D).

Generally, barley plants exposed to WS reduced their photosynthetic capacity, transpired less by closing their stomata with an overall leaves dehydration compared with their counterparts under sufficient water supply. Under WS we observed two groups of genotypes for net CO_2_ assimilation, GSW, and transpiration rate, with the two introgression lines as one, and the three elite materials as the other group (Figures 2A–C). Electron transport rate ranged from 74.49 to 179.51 μmol m^–2^ s^–1^ under WW conditions (Supplementary Figure 4). *Barke* had the lowest ETR while *S42IL-141* had the highest ETR under WW conditions. ETR was between 51.59 and 160.09 μmol m^–2^ s^–1^ under WS conditions (Supplementary Figure 4). Again, *Barke* had the least ETR, while *S42IL-143* had the highest ETR under WS (Supplementary Figure 4).

In terms of trait relationships (Supplementary Figure 5), percentage relative leaf water content was significantly (*P* ≤ 0.05) and negatively correlated with wilting score (*r* = −0.74), iWUE (*r* = −0.29), and leaf proline (*r* = −0.26). The percentage relative leaf water content significantly (*P* ≤ 0.05) and positively correlated with net CO_2_ assimilation (*r* = 0.73), GSW (*r* = 0.718), transpiration rate (*r* = 0.71), electron transport rate (*r* = 0.62), grain weight (*r* = 0.61), grain number (*r* = 0.66), plant height (*r* = 0.61), and shoot biomass (*r* = 0.36). The leaves’ susceptibility to drying, i.e., the wilting score was significant (*P* ≤ 0.05) under WS and correlated negatively with reductions in net CO_2_ assimilation rate (*r* = −0.88), GSW (*r* = −0.87), and transpiration rate (*r* = −0.88). However, leaf wilting correlated positively with leaf proline content (*r* = 0.48). The reduction in net CO_2_ assimilation rate under WS was significant (*P* ≤ 0.05) and correlated positively with reductions in GSW (*r* = 0.96), transpiration rate (*r* = 0.97), and grain weight (*r* = 0.85).

### Barley Yield Traits Under Water Stress

Grain number per main tiller had a significant genotypic, treatment, and genotype × treatment interaction effect in the 2019 experiment (Table 1). In 2018, we observed a significant (*P* ≤ 0.001) treatment effect and a genotypic effect, but no genotype × treatment interaction (Table 1). Grain number per main tiller ranged from 20 to 43 and from 2 to 24 for WW and WS treated plants, respectively (Table 2). The six-row barley, *HOR10151* had the highest number of grains per main tiller (27, 43) under WW conditions in 2018 and 2019, respectively. *S42IL-141* and *S42IL-143* had the highest grain number per main tiller (24, 24, and 22, 17) under WS conditions in 2018 and 2019, respectively (Table 2). *Barke* had the lowest grain number per main tiller (20, 6, and 24, 2) in 2018 and 2019 under WW and WS conditions, respectively (Table 2). For all genotypes, we observed at least a 30% reduction in the grain number per the main tiller under WS for the 2018 and 2019 experimental years (Supplementary Table 2).

Water stress plants showed significant variations in total grain weight per plant in both 2018 and 2019 experimental years (Table 1). We observed at least a 76% reduction in grain weight for all the genotypes investigated (Supplementary Table 2). Grain weight ranged from 9.8 to 14.0 g under WW and from 0.5 to 7 g under WS conditions (Table 2). WW *Barke* had the highest grain weight of 14 g in 2018 and 2019 (Table 2). *S42IL-141* and *S42IL-143* had the highest grain weight of 5 and 7 g under WS conditions in 2018 and 2019 (Table 2). *S42IL-141* and *S42IL-143* had more than 40% in grain weight compared with *Barke, Scarlet*, and *HOR10151* under WS (Table 2). Grain weight correlated positively with grain number per main tiller (*r* = 0.7), shoot fresh weight (*r* = 0.55), plant height (*r* = 0.76), transpiration (*r* = 0.83), GSW (*r* = 0.84), and ETR (*r* = 0.42). These correlations were significant (*P* ≤ 0.05; Supplementary Figure 5). Grain weight correlated negatively with proline (*r* = −0.49) and iWUE (*r* = −0.41). These correlations were significant (*P* ≤ 0.05; Supplementary Figure 5). WS plants had reductions of at least 38, 30, 18, and 16% in spike number, grain number, shoot fresh weight, and shoot dry weight, respectively (Table 1 and Supplementary Table 2).

Average DSI values based on the grain weight per plant ranged from 0.2 to 1.2 in 2018, and from 0.4 to 0.7 in 2019 in response to prolonged WS of 15 days, respectively (Supplementary Table 3). *Barke* had the highest DSI in both 2018 and 2019, which meant it was the most WS susceptible genotype (Supplementary Table 3). *P5cs1*-introgression line *S42IL-143*, on the other hand, had the least DSI in both 2018 and 2019 (Supplementary Table 3). Spike length had a significant genotype and genotype × treatment interaction effect in 2019 (Table 1). However, in 2018, a significant (*P* ≤ 0.01) treatment effect and a genotypic effect, were observed, with no interaction effect for spike length (Table 1). Spike length ranged from 4.5 to 10.7 cm and 6.2 to 12.7 cm under WS and WW conditions, respectively, across genotypes for both experimental years (Table 2). The spikes of the introgression lines *S42IL-143* and *S42IL-141*were the longest, both under WW and WS (Table 2). The six-row barley, *HOR10151* had the shortest spike length, both under WW conditions and WS (Table 2). Generally, WS plants had spikes that were shorter by at least 18% (Supplementary Table 2). Spearman correlation coefficient resulted in significant (*P* ≤ 0.001) and positive correlations between spike length and grain weight (*r* = 0.69), grain number (*r* = 0.54), and plant height (*r* = 0.36; Supplementary Figure 5). These data indicate that these reductions in spike length are associated with significant reductions in grain number and grain weight. Root dry weight had a significant (*P* ≤ 0.01) treatment effect and a genotypic effect, with no interaction effect for both experimental years (Table 1). The average WW root dry weight (g) was 3.9 in 2019 compared with 4.9 in 2018. The average WS root dry weight (g) was 2.9 in 2019 compared with 3.9 in 2018 (Table 1). In 2019, *Barke* had the highest root dry weight (g) of 5.75 and 5.1 under WW and WS conditions, respectively. *Scarlett* and *S42IL-141* had the lowest root dry weight (g) of 2.9 and 4.1 under WW and WS, respectively. We found no significant differences in treatment effect, genotypic, and interaction effect in root/shoot ratio (dry weight) under WW and WS in 2018 and 2019 experiments (Table 1).

### Proline Accumulation in Barley Leaves and Immature Spikes

**FIGURE 3:**
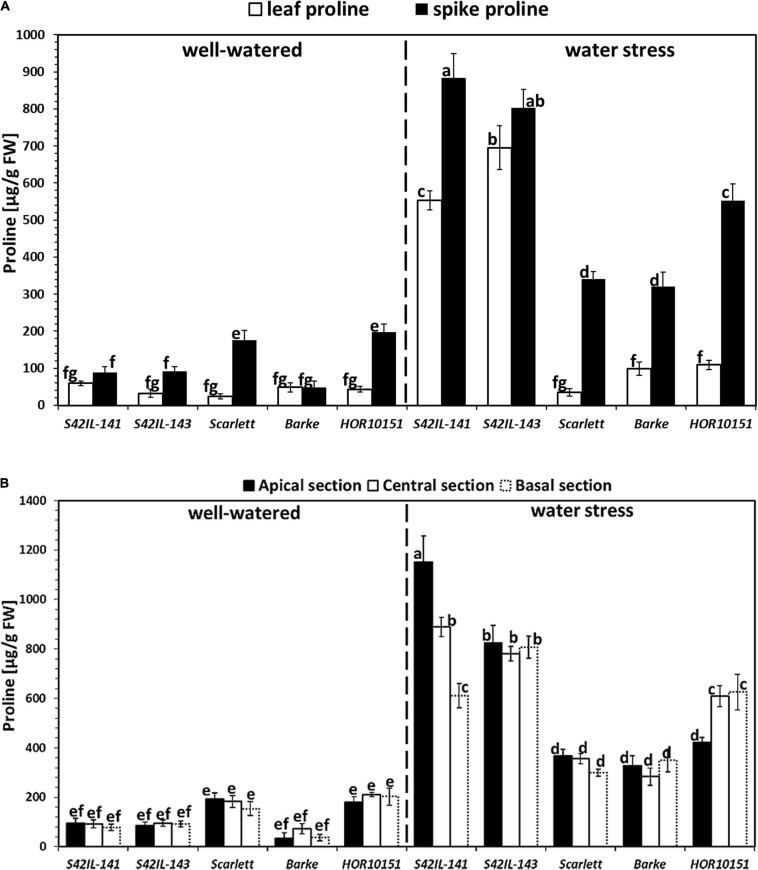
Proline accumulation to the spikes and leaves among the five barley genotypes 15 days after water stress. Different letters on the bars denote significant differences (*P* ≤ 0.05) according to Tukey’s HSD test (*n* = 6). **(A)** Proline concentration to the spike and leaf measurements for 2019 under well-watered and water stress conditions. **(B)** Spike proline concentrations along the axis of the different spike sections for the 2019 experiment under well-watered and water stress conditions.

Well-watered spike proline content ranged from 48 to 198 μg/g FW (Figure 3A). WW *Barke* and *HOR10151* had the lowest and highest spike proline, respectively. WS spike proline ranged from 319 to 884 μg/g FW (Figure 3A). Again, *Barke* had the lowest while *S42IL-141* had the highest spike proline under WS (Figure 3A). WW leaf proline ranged from 42 to 117 μg/g FW and 23 to 60 μg/g FW in 2018 and 2019, respectively (Figure 3A and Supplementary Figure 6). *S42IL-143, HOR10151*, and *Scarlett* had the lowest leaf proline under WW (Figure 3A and Supplementary Figure 6). *S42IL-141* and *Barke* had the and highest leaf proline under WW (Figure 3A and Supplementary Figure 6). Proline accumulated markedly both in the immature spikes and the leaves of barley, fifteen days after WS onset, particularly among the introgression lines (Figure 3A and Supplementary Figure 6). WS leaf proline ranged from 79 to 680 μg/g FW and 99 to 696 μg/g FW in 2018 and 2019, respectively (Figure 3A and Supplementary Figure 6). *Scarlett*, *Barke*, *and HOR10151* had the lowest leaf proline under WS (Figure 3A and Supplementary Figure 6). *S42IL-143* and *S42IL-143* had the highest leaf proline under WS (Figure 3A and Supplementary Figure 6). In detail, the immature spikes of WS introgression lines *S42IL-141* and *S42IL-143* had the highest mean proline concentrations (884 and 803 μg/g FW, respectively; Figure 3A). In contrast, immature spikes of the elite genotypes *Barke, Scarlett*, and *HOR10151* had the lowest mean proline concentrations (319, 341, and 552 μg/g FW, respectively) under WS (Figure 3A). *HOR10151* and *Scarlett*, compared with the other three genotypes, exhibited an increase of about 198 μg/g FW of spike proline under WW (Figure 3A). Leaf proline concentrations in the genotypes expressed per unit dry weight (DW) showed significant (*P* ≤ 0.001) differences as well (Supplementary Table 1), which followed a similar trend to the proline concentrations measured per unit FW (Figure 3A and Supplementary Table 1). Leaf proline per dry biomass ranged from 5 to 14 μmol/g under WW and 5 – 55 μmol/g under WS (Supplementary Table 1). The introgression line *S42IL-143* had the highest proline concentration on a dry biomass basis, and the elite genotype, *Scarlett*, the lowest (Supplementary Table 1).

In the experiment of 2019, proline from the basal, central, and apical sections of the immature spikes showed significant differences among the genotypes and treatments (Figure 3B). WW spike proline from the apical, central, and basal sections ranged from 34, 72, and 37 to 192, 211, and 202 μg/g FW, respectively (Figure 3B). WW spike proline of *Barke* (34, 72, and 37 μg/g FW) from the apical, central, and basal sections, respectively, was the lowest (Figure 3B). The spike proline of the apical section of *Scarlett* (192 μg/g FW), was the highest among all the five genotypes under WW conditions. Spike proline from the central and basal sections of *HOR10151* (211 and 202 μg/g FW) was the highest under WW conditions (Figure 3B). WS spike proline from the apical, central, and basal sections ranged from 327, 283, and 299 to 1151, 889, and 806 μg/g FW, respectively (Figure 3B). WS spike proline of *Barke* (327 and 283 μg/g FW) from the apical and central sections, respectively, were the lowest while *Scarlett* the lowest for the basal section (Figure 3B). Spike proline from the apical and central sections of *S42IL-141* (1151 and 889 μg/g FW) was the highest under WS conditions (Figure 3B). WS spike proline from the basal section of *S42IL-143* (809 μg/g FW) was highest among all five genotypes (Figure 3B).

The distribution of proline along the spike was not uniform for any of our barley genotypes under WS (Figure 3B). For instance, we found an increase of at least 40% in proline among the apical and central spike sections of *S42IL-141* (1152 and 889 μg/g FW) compared with *HOR10151* (422 and 609 μg/g FW), respectively (Figure 3B). This increase under WS did not follow a clear position-dependent gradient along the spike, although the introgression lines generally had at least a 10% higher spike proline (Figure 3B). There were no differences in proline content in the basal spike section of *S42IL-141* (612 μg/g FW) to the basal and central section of *HOR10151* (626 and 609 μg/g FW) under WS (Figure 3B). In summary, section-specific differences existed considering the apical, central, and basal spike proline of *S42IL-141* and *HOR10151* individually under WS.

Analysis of spike and leaf revealed a higher increase in proline concentration in the spikes than in the leaves for all genotypes under WS (Figures 3A,B). *P5cs1-*introgression lines had a significantly higher proline concentration in their developing spikes than the leaves under WS conditions, exhibiting an average difference of 30% (Figure 3A). Similarly, elite genotypes *Barke, Scarlett*, and *HOR10151* also had markedly more proline in their immature spikes than in the leaves under WS conditions (average difference of 134%, Figure 3A). However, in absolute terms, the introgression lines had higher spike proline content than the elite lines under WS (Figures 3A,B).

### Imaging of Water-Stressed Spikes With MRI

**FIGURE 4:**
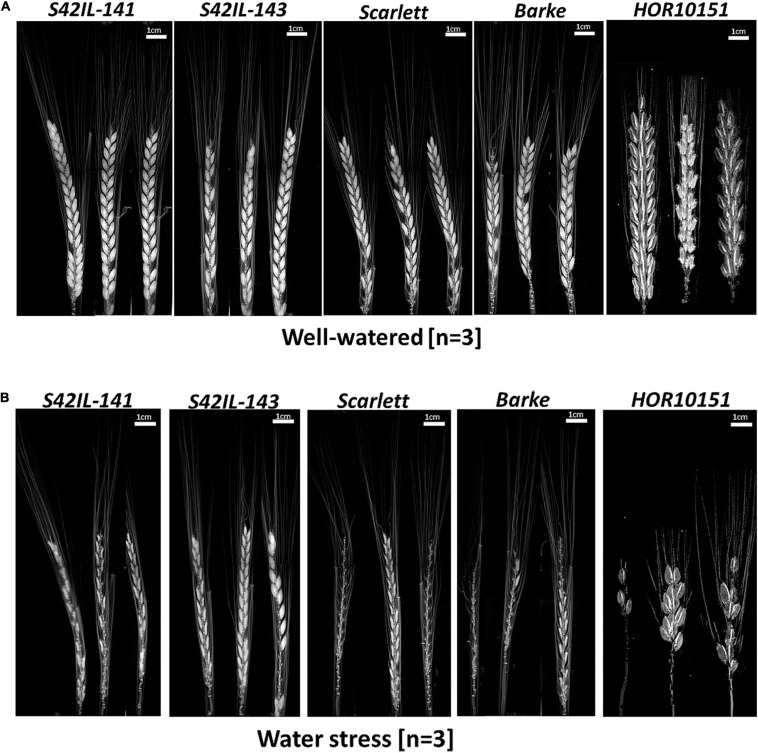
MRI amplitude images of barley main spikes at BBCH-scale 83, 15 days after stress application. **(A)** Main spikes of *S42IL-141, S42IL-143, Scarlett, Barke*, and *HOR10151* grown under well-watered conditions. **(B)** Spikes from plants grown under water stress (*n* = 3, scale = 1 cm).

To examine the effect of WS on seed abortion and filling early in the reproductive development phase (before grain maturation) of barley, we used MRI to scan immature spikes at the BBCH-scale, 83, i.e., at the soft milky dough stage (Figures 4A,B and Supplementary Figure 7). We acquired amplitude images of 2D projections of barley spikes and evaluated them for the presence of initiated, developing, fully developed, sterile, or aborted seeds (Figures 4A,B and Supplementary Figure 7). We did MRI scans of intact spikes at the early dough stage. Seed abortion was more prevalent among the elite genotypes (*Barke*, *Scarlett*, and *HOR10151*) than in the introgression lines (*S42IL-143* and *S42IL-141*) after prolonged 15 days of WS treatment (Figures 4A,B). Poor seed yield performance among the elite lines compared with the introgression lines under WS (Tables 1, 2), were additionally revealed by several of our phenotypic traits (spike length, grain number, grain weight) similar to the MRI observations (Figures 4A,B). MRI scans (Figure 4A) of WW spikes of all genotypes showed a lower seed abortion rate, or no abortion at all, for all our barley types (Figure 4A). MRI scans of whole spikes grown under prolonged WS treatment, however, showed increased seed abortion (and in some cases complete spike abortion) among the elite genotypes, *Scarlett*, *Barke*, and *HOR10151*, much more so than the introgression lines, *S42IL-143*, and *S42IL-141* (Figure 4B). For all genotypes, WS-treated main spikes were found to contain shriveled or small developing grains (Figure 4B). Conversely, none of the spikes from WW plants showed shrunken seeds (Figure 4A).

Grain filling under WS thus was reduced more among the elite genotypes than in the introgression lines (Figure 4B and Table 2). Also, under WS grain number in *Scarlett*, *Barke*, and *HOR10151* was reduced more than in *S42IL-143* and *S42IL-141* (Figure 4B). These results were confirmed by the 2019 seed count (Table 2). At harvest, under WS the grain numbers of the main spike of *S42IL-143* and *S42IL-141* were 17 and 22, respectively; significantly higher than for the elite genotypes of *Scarlett*, *Barke*, and *HOR10151* (9.5, 2, and 4, respectively) (Table 2). Again, similar to what we observed in the MRI projections (Figures 4A,B), phenotypic spike length of *S42IL-143* and *S42IL-141* were significantly longer than *Scarlett*, *Barke*, and *HOR10151* under WS (Tables 1, 2). In summary, both MRI and phenotypic data confirmed that the introgression lines performed better in terms of seed yield than the elite lines under WS.

## Discussion

We characterized barley *P5cs1-*introgression lines and their physiological responses to reduced water availability. To consolidate our findings, we conducted two experiments in a greenhouse with a genotype panel including the same introgression lines and elite cultivars and with the same type of WS treatment applied at pre-flowering stages and kept as constant as possible throughout spike and seed development. Calculation of thermal sums for the whole duration of the two experiments shows that there was a difference of only about 5% in cumulated degree-days between the experiments of 2018 and 2019 (see section “Materials and Methods”). However, average daily temperatures were generally lower (i.e., below 25°C) during and after flowering time in 2019 compared with 2018. DLI maximum values were very similar in both experimental years whereas the minimum values were generally lower in 2018 compared with 2019 (Supplementary Figure 1). Overall, under WW conditions we obtained very similar results in the two subsequent experimental years (Supplementary Table 2). We note that average grain weight per plant at harvest under WW conditions was somewhat higher in 2019 compared with 2018, which might be linked to the lower daily temperatures during GF. Imposing WS conditions at booting stages resulted in overall more severe effects in 2019 compared with 2018, in particular leading to a more pronounced decrease in tiller numbers and shoot weight at harvest on average, considering all genotypes (Supplementary Table 2). In addition, relative water content and net assimilation rates measured three days after the onset of the drought treatment were also lower in 2019 compared with 2018. Because these effects cannot be simply explained by temperature and DLI differences between the two years, we conclude that the first few days after the onset of WS led to a more pronounced decrease in soil water content values in 2019, as we can observe by comparing the time profile of pot soil moisture measured by time-domain-reflectance sensors (Supplementary Figure 2).

Drought is a complex trait and may lead to several morpho-physiological alterations within a plant. As an adaptation to drought stress, plants adjust their transpiration, photosynthesis, and thus WUE, to prevent water loss and tissue damage while preserving the capacity for CO_2_ assimilation (Belko et al., 2012; Li et al., 2017). Naz et al. (2014) and Muzammil et al. (2018) reported earlier on the same introgression lines, *S42IL-143*, and *S42IL-141* that they maintained 70% percentage relative leaf water content and displayed less severe wilting under WS. These findings were confirmed in our study. *S42IL-143* and *S42IL-141* maintained relative leaf water content of more than 70% even under WS (Table 2). On average, iWUE of the introgression and elite lines under WS increased by 20 and 7%, respectively. The two introgression lines often had very similar responses in terms of net CO_2_ assimilation, GSW, and transpiration rate under WS (Figures 2A–C). Yang et al. (2019) reported similar increases in iWUE (39 and 37%) for their contrasting rice hybrid cultivars under drought. WS caused an increase in the duration of spike development of all our barley genotypes. Earlier studies (Matin et al., 1989; Rani et al., 2018), reported a prolonged duration in spike development due to WS at GF. The impact of WS on plant floral development generally might either cause a shortened or prolonged life cycle taking into account genotype specificity leading to an overall reduction in productivity (Dolferus, 2014; Boussora et al., 2019). Similarly, Li et al. (2017) reported wide variations in gas exchange parameters of drought-treated flag leaves compared with control conditions at the heading stages of drought-susceptible wheat cultivars compared with tolerant genotypes.

Water shortage during the post-anthesis period has been shown to significantly reduce harvest index and grain yield (Vadez et al., 2014). Due to the dehydrating effect of the WS treatment, 15 days after stress the seeds of all genotypes became smaller (Figure 4B). All spikes showed strong reductions in spike length, seed size and number under WS conditions (Figure 4B). Consequently, there was a significant loss of 76% in total grain weight (all genotypes averaged; Table 1). The elite genotypes *HOR10151* or *Scarlett* and *Barke* showed pronounced leaf wilting symptoms, leaf dehydration, and significant seed abortion overtime under WS (Table 2 and Figures 1, 4A,B). *IL143* and *IL141* on the other hand showed less wilting symptoms and less leaf dehydration (Figure 1). These effects were also reflected in the spikes values. Grain number and size of the introgression lines were also severely affected by WS but performed better than the elite genotypes (Figures 4A,B). These results are similar to studies obtained with computed tomography of wheat grains under WS or heat treatment, which showed shriveled seeds in 3D projections (Tracy et al., 2017; Schmidt et al., 2020). These findings confirm that low water use during the post-anthesis period significantly reduces both harvest index and grain yield (Vadez et al., 2014) and further highlight the critical importance of maintenance of plant water status before and during the grain-filling period.

Prior studies have noted the importance of proline accumulation in many plant species, as one of the most prominent changes in plant metabolism during drought and low soil water potential (Shinde et al., 2016). Contrary to proline accumulation in reproductive organs, proline accumulation in leaves and roots has been extensively researched in earlier work (Verbruggen and Hermans, 2008). Proline accumulation in different plant organs is time-dependent and different concentrations have been reported for different plant species even under apparently similar stress scenarios. Proline accumulates rapidly and is degraded as the plant recovers (Dar et al., 2016; Heuer, 2016). Mickky et al. (2019) reported an increase in leaf proline content in ten wheat cultivars under drought conditions. They observed that proline accumulation was more pronounced in the drought-tolerant cultivars than in the sensitive ones. Similarly, in our investigation, we identified a more than fivefold increase in leaf proline under WS in the tolerant introgression lines *S42IL-143* and *S42IL-141*, much higher than in the susceptible elite genotypes *Barke*, *Scarlett*, and *HOR10151* (Figures 3A,B). In a similar study, Templer et al. (2017) found a more than fivefold increase for leaf proline content under drought and heat stress in their tolerant barley genotypes, as compared with control.

In the current study, we report higher proline contents in the reproductive structures of the WS immature spikes than in the leaves, for all our barley genotypes. Accumulation of proline in undeveloped seeds of *Vicia faba* indicated that proline might play an essential role in the development of generative organs (Venekamp and Koot, 1984). Numerous studies reported high-proline contents in Arabidopsis seeds developing under WS (Chiang and Dandekar, 1995; Schmidt et al., 2007), although data on proline accumulation in seeds of other species are more scarce (Dar et al., 2016). In our study, *P5cs1*-introgression lines accumulated the highest proline amounts (+30%) in their developing spike compared with leaves of elite genotypes (Figure 3A). These elite genotypes under WS had more than double the proline content in their immature spikes than in their leaves (Figure 3B).

Proline accumulation is a common physiological response to various stresses but is also part of the developmental program in generative tissues (Heuer, 2016). Proline may act as an osmoprotectant to protect the actively growing cellular and subcellular structures of the spike from dehydration under WS (Chiang and Dandekar, 1995). Further evidence suggests that proline is also involved in flowering and development both as a metabolite and possibly as a signal molecule (Dar et al., 2016). In our introgression lines, higher proline content was associated with higher relative leaf water content and a reduced wilting score (Table 1). As a protective mechanism to WS, barley, and wheat are known to allocate proline to actively growing vegetative tissues in shoots and roots. This is associated with reduced dehydration and wilting under WS (Delauney and Verma, 1993; Lee et al., 2009; Bandurska et al., 2017; Koenigshofer and Loeppert, 2019). In barley, Cai et al. (2020) reported that genotypes that show less leaf wilting under stress were able to osmotically adjust and better tolerate water shortage. In our study *P5cs1*-introgression lines showed less severe leaf wilting (−40%) under WS compared with elite counterparts, which indicates reduced susceptibility to soil drying conditions.

We found a higher ETR in the drought-tolerant introgression *S42IL-143* under WS than in the elite lines. As Shinde et al. (2016) emphasized, proline metabolism regenerates NADP^+^ to provide a continued supply of electron acceptors for chloroplast electron transport. However, most drought susceptible genotypes like *Scarlett* fail to accumulate and use proline because of early leaf wilting and leaf death, resulting in proline reduction under drought conditions (Sayed et al., 2012). We found a higher net CO_2_ assimilation rate, reduced transpiration, stomatal opening, intrinsic water use efficiency, and an active ETR several days after WS in *P5cs1-S42IL-143* and *S42IL-141*compared with *Barke*, *Scarlett*, and *HOR10151* under WS (Figures 2A–D and Supplementary Figure 4). The net CO_2_ assimilation rate of *S42IL-143* and *S42IL-141*was more than double the rate of *Barke*, *Scarlett*, and *HOR10151* under WS. *S42IL-143* and *S42IL-141*had a marginally (5%) higher GSW compared with the elite lines *Barke*, *Scarlett*, and *HOR10151* under WS. A contributing factor to the higher GSW and overall photosynthetic rate of the introgression lines under WS is their wild allele *P5cs1*. It has been shown to enhance the drought protective mechanism of proline biosynthesis (Szabados and Savouré, 2010; Sucre and Suárez, 2011; Allahverdiyev, 2015; Qamar et al., 2015). Several reports have already established that drought-tolerant barley genotypes accumulate proline to maintain GSW and active photosynthesis, even under dehydrating conditions, while drought-sensitive genotypes immediately reduce the stomatal aperture (Deng et al., 2013; Marok et al., 2013; Naz et al., 2014; Haddadin, 2015).

In the current study, our introgression lines achieved approximately double the grain weight of the elite lines under WS. Similar results were reported by Templer et al. (2017), who under drought conditions found a decrease of 65% in the harvest index in drought susceptible German cultivars, whereas drought-tolerant Mediterranean cultivars decreased not more than 14%. Based on a DSI which we calculated from the grain weight per plant, the most tolerant genotype was *S42IL-143* with the least DSI of 0.2, 0.4 in 2018, and 2019, respectively after fifteen days of WS (Supplementary Table 3). Haddadin (2015), reported a DSI of >1 for susceptible spring barley and <0.5 for tolerant types. A possible explanation for the higher grain yield under WS by *S42IL-143* and *S42IL-141*is the enhanced proline accumulation (Sayed et al., 2012). The observed proline increases due to WS also had significant correlations with reduced grain number, grain yield, plant height, and shoot biomass (Supplementary Figure 5). Sallam et al. (2018) previously reported an increase in grain proline and reduction in starch content due to heat stress, with significant reductions in yield per plot, grain yield per spike, and 1,000-kernel weight. Several studies reported a negative correlation between shoot proline concentration, growth and yield traits (Bandurska et al., 2017; Boussora et al., 2019). However, this negative correlation might be interpreted as an indication that the plants experienced WS, and not necessarily reflect a causal relation between proline accumulation and reduced plant growth and yield. On the contrary, in our study, we found drought-induced proline accumulation in the spikes of barley genotypes harboring the wild variant of *P5cs1* to be associated with improved drought tolerance, as expressed in their photosynthetic capacity, seed number, and final yield under WS.

Proline is a highly inter-convertible organic molecule. It is transiently up regulated to tackle the effect of drought stress, but can also be catabolized to energy rich metabolites as soon as water availability improves. It thus has multiple roles in drought stress adaptation and stress recovery (Szabados and Savouré, 2010; Forlani et al., 2019). Proline is likely associated with the energy demand of young dividing cells during resumed growth following stress relief (Verslues and Sharma, 2010). This possibility is also corroborated by findings at the transcriptional level in which *P5CS2*, *P5CR* (encoding *P5C* reductase), and *ProDH1* are upregulated in meristematic tissues such as root tips, shoot apices, lateral buds, and the inflorescence (Sharma et al., 2011). Young spike tissues of all our genotypes at their early grain development of the soft milky dough stages had accumulated more proline. Most root and leaf cells that are actively dividing, elongating, and developing also tend to accumulate proline under drought (Dar et al., 2016), which contributes to coping with drought stress during reproductive development and to increasing proline sink strength in those tissues (Kishor and Sreenivasulu, 2014). The question of where proline synthesis primarily occurs in plants upon imposition of stress still remains to be clarified. Proline metabolism varies among organs and tissues, and transport of proline within the plant is likely to occur (Koenigshofer and Loeppert, 2019). Aside from the relevance of proline in stress adaptation, the effect of higher proline content on grain quality need to be investigated comprehensively before utilizing these genetic resources in plant breeding. Therefore, in future work we will investigate the effect of proline mediated stress adaptation on grain quality, total protein content as well as on brewing quality traits.

## Conclusion

Prolonged WS at the booting stage caused a significant reduction of 76% of barley grain weight per plant. We found drought-inducible proline accumulation to be not exclusive to the leaves, rather proline significantly accumulates in barley spikes and it may contribute to the maintenance of seed initiation and GF processes by preventing excessive water loss. Spike proline content under WS increased by more than 30% compared with leaf proline content in all our barley genotypes. *P5cs1*-introgression lines harboring a wild barley allele involved in the proline biosynthetic pathway had higher leaf and spike proline contents as well as a higher grain yield under WS conditions. Generally, the elite lines were much more affected by WS than the introgression lines on several morpho-physiological traits. *S42IL-143* and *S42IL-141* carrying the *P5cs1* allele from wild barley showed an increased WS tolerance associated with a reduced seed abortion rate and a higher spike proline concentration compared with Scarlett, *Barke*, and *HOR10151*. Our results suggest that proline accumulation in spikes of barley under WS plays a major role in the maintenance of final seed yield. Future studies will focus on the validation of presented physiological variation in field conditions as well as to evaluate the effect of elevated proline on grain quality traits.

## Data Availability Statement

The original contributions presented in the study are included in the article/Supplementary Material, further inquiries can be directed to the corresponding author.

## Author Contributions

FFr, CW, DD, AN, MF, and FFi conceptualized and designed the research. FFr conducted the experiments, analyzed the data, and wrote the manuscript. CW, DD, AN, MF, and FFi contributed to writing the manuscript. CW, DD, and FFi supervised the research. All authors approved the submitted version.

## Conflict of Interest

The authors declare that the research was conducted in the absence of any commercial or financial relationships that could be construed as a potential conflict of interest.

## Footnotes

1 http://www.varianinc.com

## Abbreviations

*A*: net CO_2_ assimilation
B: booting
DAWS: days after water stress
DLI: daily light integral
DSI: drought susceptibility index
*E*: transpiration rate
ETR: electron transport rate
FW: fresh weight
GF: grain filling
GSW: stomatal conductance
HD: heading
MRI: magnetic resonance imaging
PPFD: photosynthetic photon flux density
*P5cs1*: *pyrroline-5-carboxylate synthase1*
*P5CS2*: *pyrroline-5-carboxylate synthase2*
*P5CR*: *pyrroline-5-carboxylate reductase*
*P5C*: *pyrroline-5-carboxylate*
*ProDH1*: *proline dehydrogenase1*
WS: water stress
WW: well-watered.
