# Supplementary material for: A Wild Allele of Pyrroline-5-Carboxylate Synthase1 Leads to Proline Accumulation in Spikes and Leaves of Barley Contributing to Improved Performance Under Reduced Water Availability
Source: Front Plant Sci. 2021 Feb 25;12:633448. doi: 10.3389/fpls.2021.633448 (PMC7947243; doi:10.3389/fpls.2021.633448)
Supplement: Supplementary file 1 [file Data_Sheet_1.pdf]

Supplemental Table S1. Estimated proline concentration on a dry biomass basis (2019)

Treatment	Genotype	Shoot dry biomass/plant (g)	Proline content (amount/plant, µmol/g)
	S42IL-141	14.68	7.63 **d**
	S42IL-143	16.72	5.40 **d**
**Well-watered**	*Scarlett*	16.06	5.73 **d**
	Barke	26.42	12.17 **c**
	HOR10151	20.58	14.29 **c**
	S42IL-141	11.37	54.70 **b**
	S42IL-143	13.64	81.46 **a**
**Water stress**	*Scarlett*	10.83	5.39 **d**
	Barke	15.70	15.29 **c**
	HOR10151	9.17	12.29 **c**

The estimated content proline was calculated based on the mean dry biomass multiplied by the metabolite concentration (Podda et al., 2019). The different letters a-d represents Tukey's HSD test of significance ($P \leq 0.001$).

Supplemental Table S2. Range of variation and relative percentage change of morphological, yield, and physiological traits under well-watered (WW) and water stress (WS) conditions during the 2018 and 2019 experimental years. Morphological and yield traits were measured at harvest, photosynthesis and gas exchange parameters were measured three days after the onset of water stress.

	Data range (min-max)				Relative percentage change (100 × WS -WW/WW)	
	WW		WS			
**Selected plant trait**	**2018**	**2019**	**2018**	**2019**	**2018**	**2019**
Plant height (cm)	77-105	79-106	59-88	61-82	-18	-27
Tiller number	14-23	16-25	10-19	6-17	-19	-47
Spike number	16-22	21-30	6-12	11-22	-45	-38
Length of main spike (cm)	6-14	6-13	4-12	3-13	-18	-22
Grain number per main tiller	14-43	15-61	0-28	0-30	-30	-58
Grain weight (g)	7-8	8-18	1-8	0-9	-76	-76
shoot fresh weight (g)	23-95	16-116	16-69	5-28	-18	-44
% Relative leaf water content	82-94	76-97	50-92	14-91	-15	-35
Net CO_2 Assimilation (µmol m^{-2} s^{-1})	20-30	20-22	8-22	3-10	-56	-72
Stomatal conductance (mol m^{-2} s^{-1})	0.33-0.55	0.20-0.40	0.069-0.19	0.02-0.10	-74	-77
iWUE (µmol CO_2 $mmol^{-1}$ H_2O)	55-82	61-94	87-149	69-111	+73	+17
Electron transport rate (µmol m^{-2} s^{-1})	130-194	127-180	70-134	51-152	-31	-28
Transpiration rate (mol m^{-2} s^{-1})	$8.0E^{-3}$-$1.2E^{-2}$	$4.0E^{-3}$-$9.0E^{-3}$	$1.9E^{-3}$-$5.9E^{-3}$	$5.2E^{-4}$-$3.0E^{-3}$	-63	-76

Supplemental Table S3. A drought susceptibility index (DSI) was calculated based on total grain weight per plant (g) at harvest for all genotypes and years.

Year	***Genotypes***	***DSI***
	Barke	1.2
	HOR10151	1
**2018**	*Scarlett*	0.6
	S42IL-141	0.26
	S42IL-143	0.25
	Barke	0.7
	HOR10151	0.63
**2019**	*Scarlett*	0.63
	S42IL-141	0.5
	S42IL-143	0.4

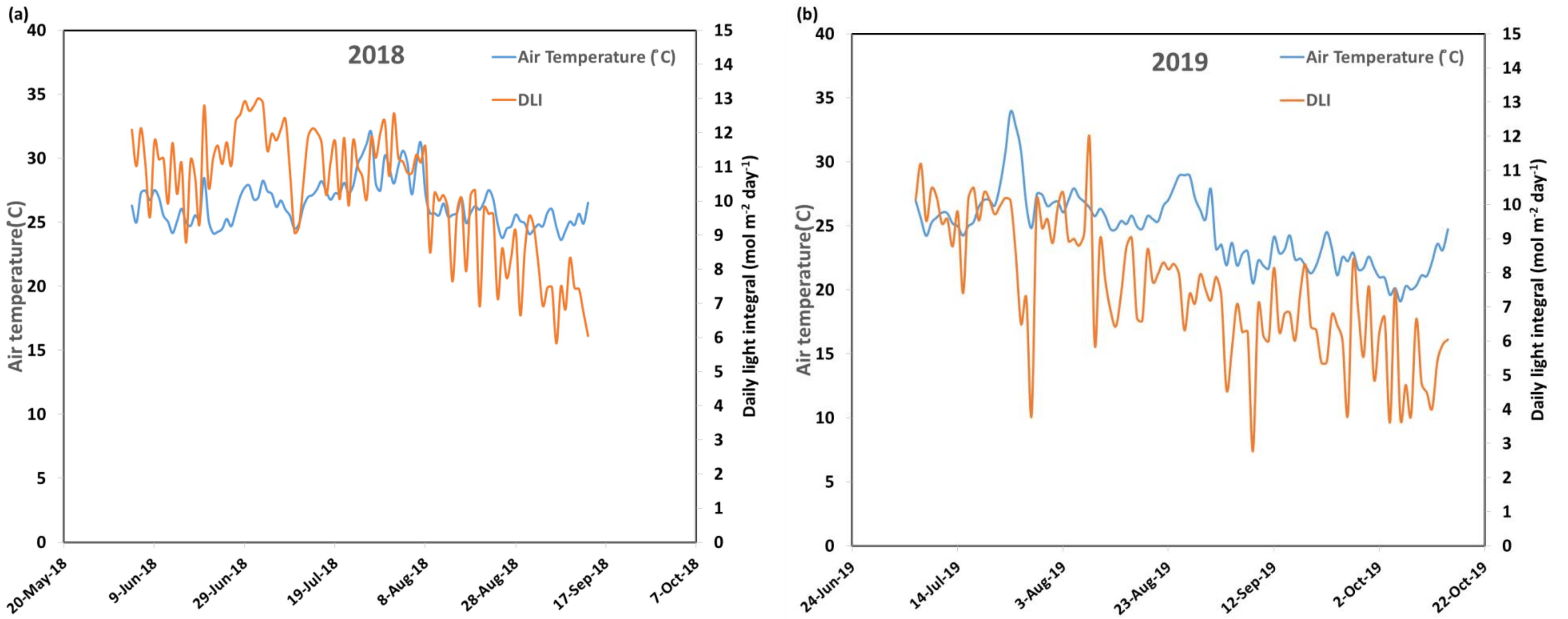

Supplemental Figure S1. Daily mean air temperature (℃) and daily light integral (mol m^{-2} day^{-1}**)** recorded at the greenhouse during the experiments in 2018 (a) and 2019 (b).

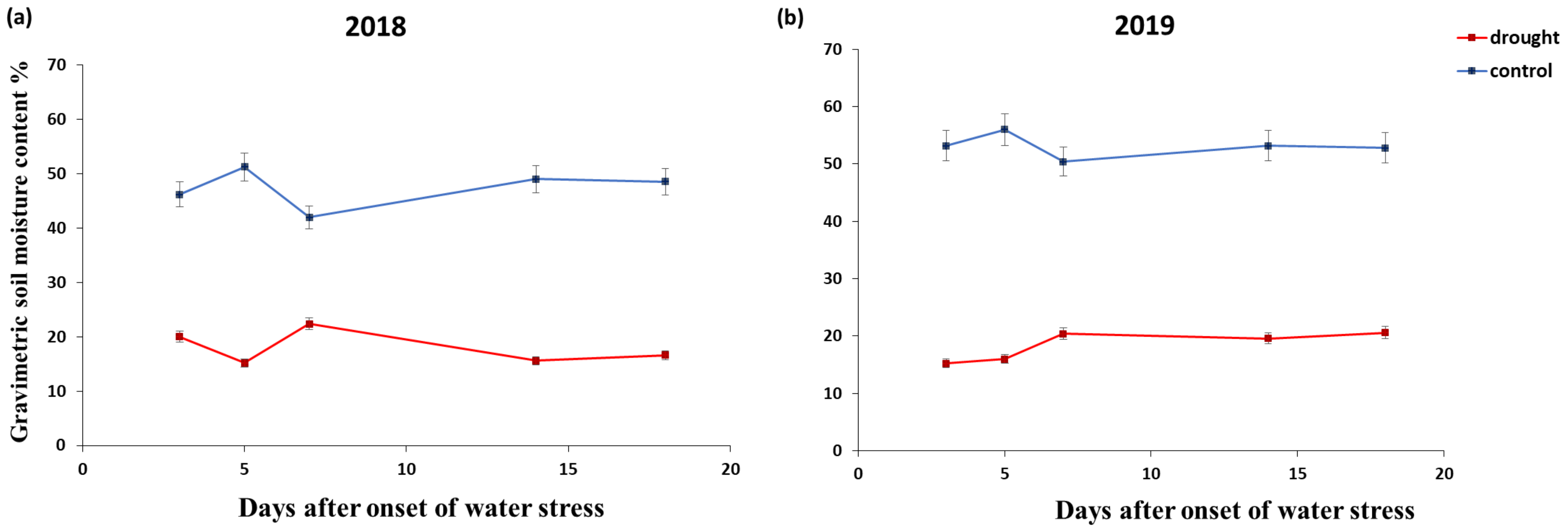

Supplemental Figure S2. Line graphs of the gravimetric soil moisture content measured with the Theta ML2 probe during the application of the two irrigation regimes for 2018 (A) and 2019 (B) experiments. Blue-line represents the percentage gravimetric moisture content of the well-watered plants (~50% g/g) and the red line is the percentage gravimetric moisture content of the water stress plants after two days of dried down (water stress ~20% g/g).

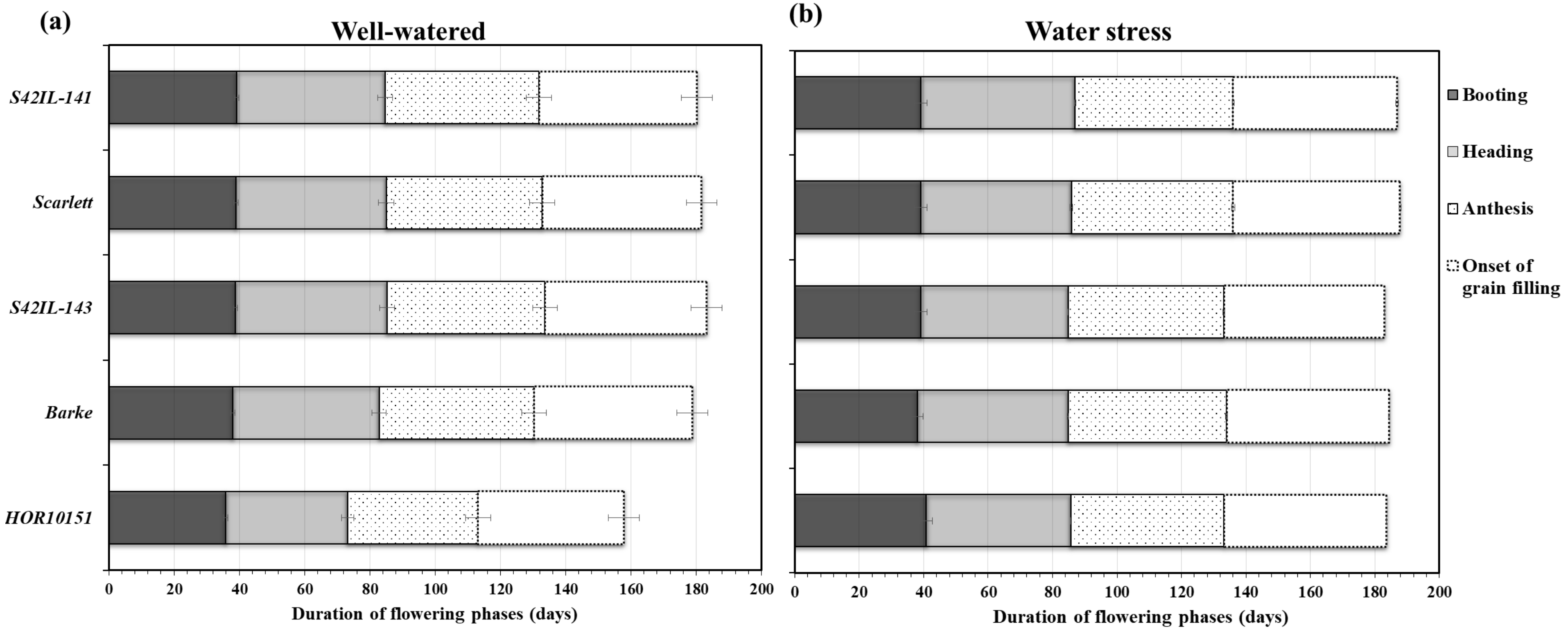

Supplemental Figure S3. Duration of flowering phases of the spikes under well-watered condition (A) and water stress conditions (B). The legend indicates the various spike developmental stages from booting, heading, anthesis, and on-set of grain filling. The Y-axis shows the different genotypes.

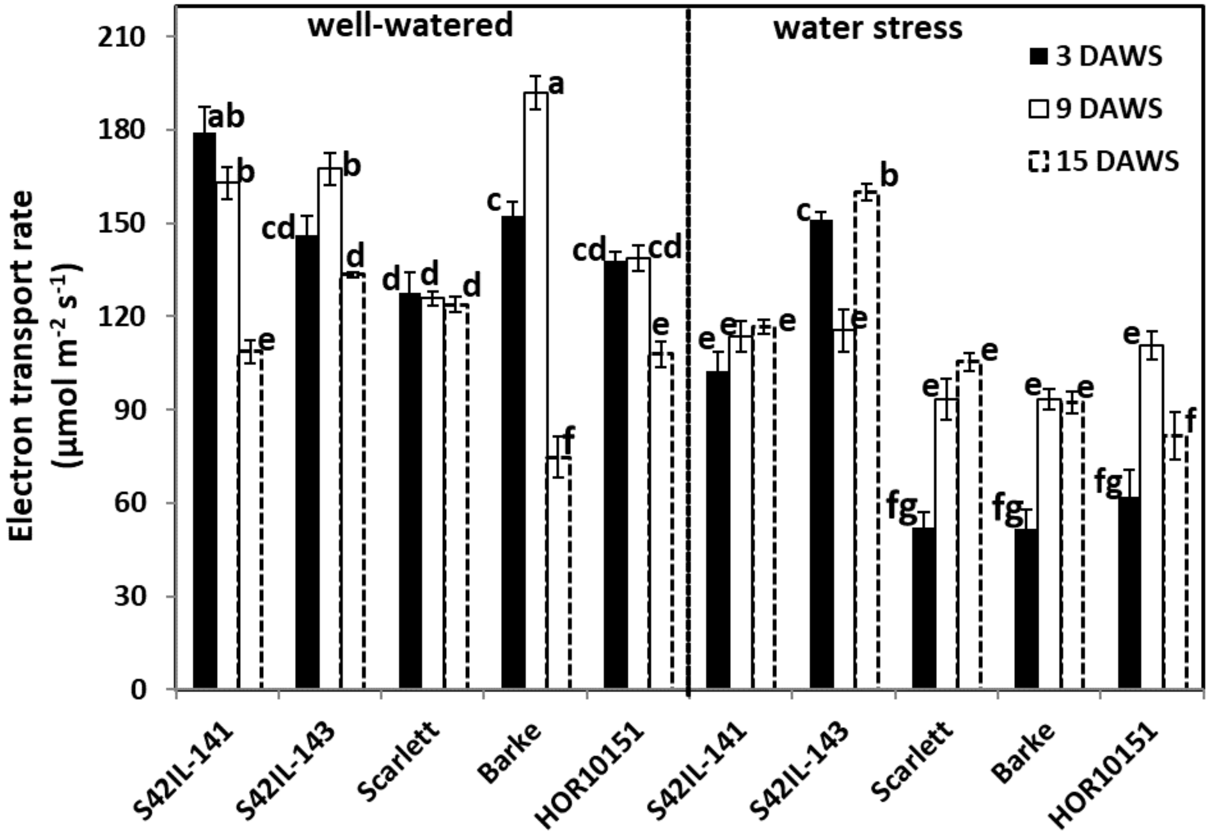

Supplemental Figure S4. Bar plot of electron transport rate, y-axis, for the different barley genotypes under well-watered and water stress treatments, the x-axis is the different genotypes. The legend represents the measurement days of 3, 9, and 15 days after drought stress (DAWS) *i.e.* at booting, heading, and on-set of grain filling stages of floral development. Different letters on the bars denote significant differences ($P \leq 0.05$) according to Tukey's HSD test.

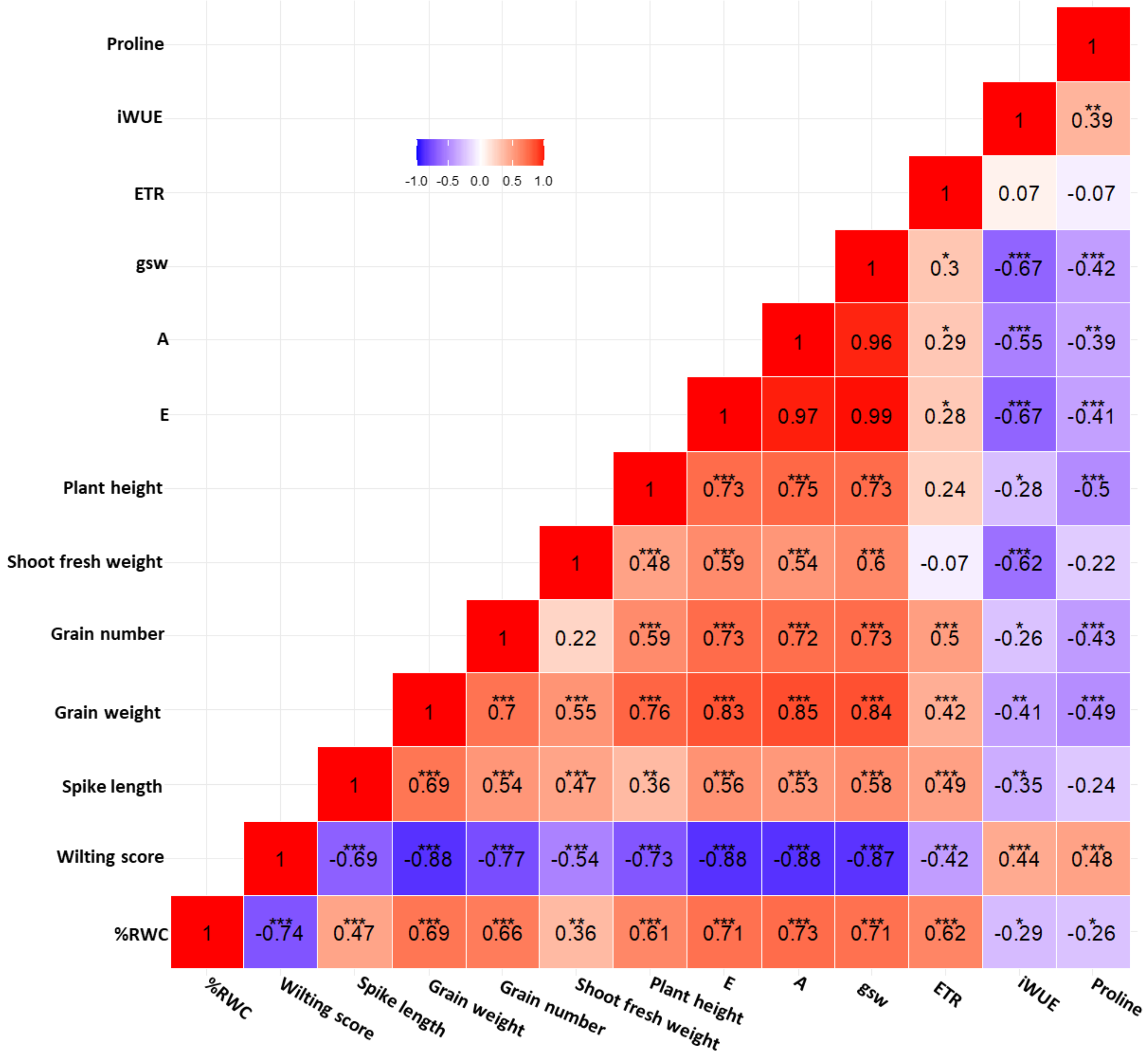

Supplemental Figure S5. Spearman correlation heat map of selected plant traits for pairwise comparison based on our 2019 data. Significant correlations "*, **, ***" follows the standard probability values ($P \leq 0.05$, $P \leq 0.01$or $P \leq 0.001$). A: Net CO_2 assimilation, E: transpiration, gsw: stomatal conductance, % RWC: percentage relative leaf water content, iWUE: intrinsic water use efficiency (A/gsw), and ETR: electron transport rate.

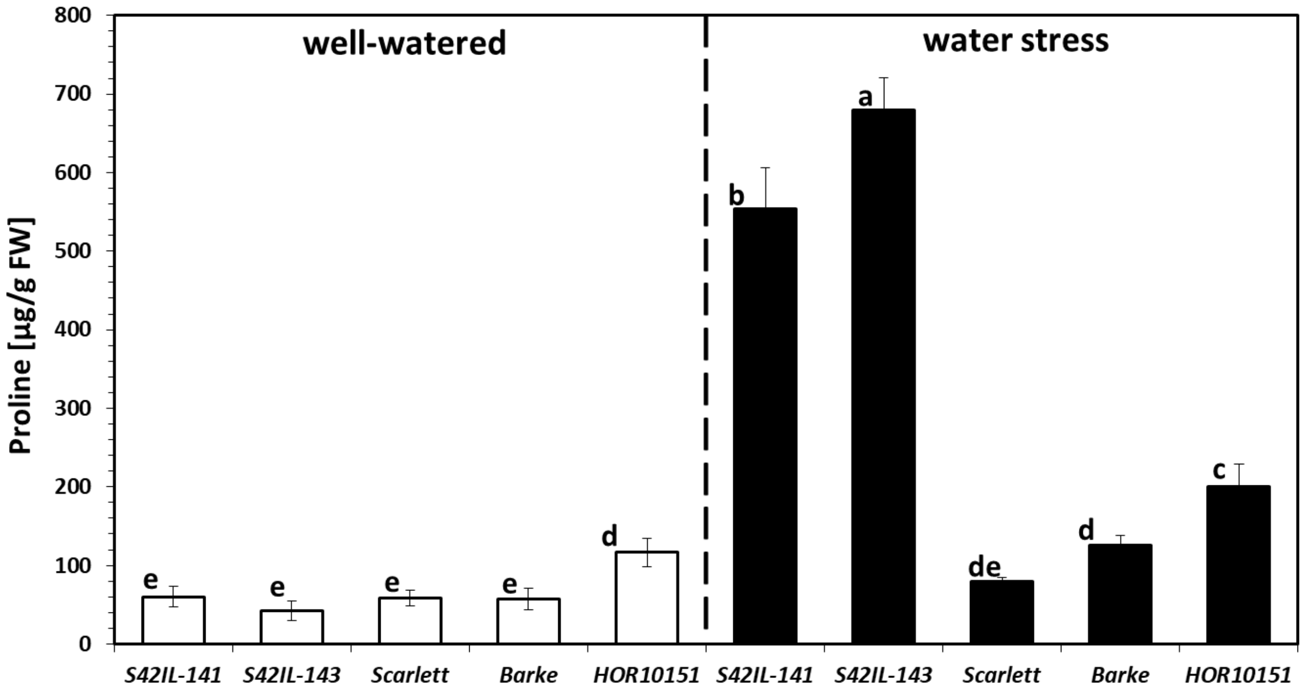

Supplemental Figure S6. Leaf proline for 2018 under well-watered and water stress conditions for the different barley genotypes. Different letters on the bars denote significant differences ($P < 0.05$) according to Tukey's HSD test.

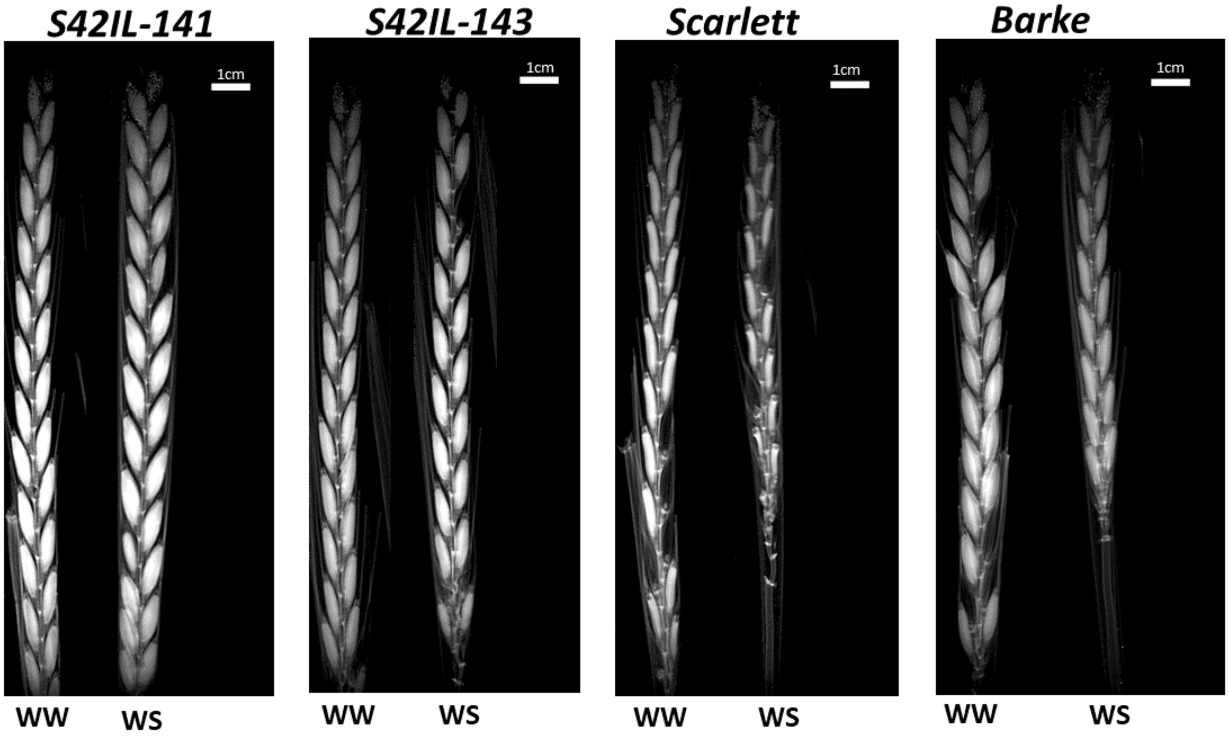

Supplemental Figure S7. 2018 MRI amplitude images with multiple spin-echo sequence of single slices of barley whole intact main spikes at BBCH 83, 15 days after stress application. n=1, scale = 1cm.
