# Supplementary material for: A Wild Allele of Pyrroline-5-Carboxylate Synthase1 Leads to Proline Accumulation in Spikes and Leaves of Barley Contributing to Improved Performance Under Reduced Water Availability
Source: Front Plant Sci. 2021 Feb 25;12:633448. doi: 10.3389/fpls.2021.633448 (PMC7947243; doi:10.3389/fpls.2021.633448)
Supplement: Supplementary file 2 [file Data_Sheet_2.pdf]

Three way ANOVA
Sources of variation = treatment:genotype:year
```
**********************************************************************
********************************************************
**********************************************************************
********************************************************
```

1. %Relative leaf water content

Shapiro-Wilk normality test

```
data:  resRWC
W = 0.99582, p-value = 0.8398

summary(anovaRWC)
                         Df Sum Sq Mean Sq F value   Pr(>F)    
treatment                 1  33696   33696 1088.91  < 2e-16 ***
genotype                  4  20656    5164  166.88  < 2e-16 ***
year                      1   5527    5527  178.59  < 2e-16 ***
treatment:genotype        4  24349    6087  196.71  < 2e-16 ***
treatment:year            1   3114    3114  100.64  < 2e-16 ***
genotype:year             4   2078     519   16.79 8.44e-12 ***
treatment:genotype:year   4   1310     327   10.58 9.24e-08 ***
Residuals               190   5880      31                     
---
Signif. codes:  0 â€˜***â€™ 0.001 â€˜**â€™ 0.01 â€˜*â€™ 0.05 â€˜.â€™ 0.1
â€˜ â€™ 1
> print(model.tables(anovaRWC,"means"),digits=3)
Tables of means
Grand mean
         
72.82724 

 treatment 
treatment
control drought 
   85.5    60.2 

 genotype 
genotype
    Barke  HOR10151 S42IL-141 S42IL-143  Scarlett 
     61.8      60.9      81.0      85.3      75.0 

 year 
year
2018 2019 
80.9 69.6 

 treatment:genotype 
         genotype
treatment Barke HOR10151 S42IL-141 S42IL-143 Scarlett
  control 88.0  85.1     84.5      85.0      84.8    
  drought 35.5  36.8     77.5      85.7      65.3    
```

```
 treatment:year
         year
treatment 2018 2019
  control 87.5 84.7
  drought 74.4 54.5

 genotype:year
           year
genotype    2018 2019
  Barke     72.9 57.3
  HOR10151  71.8 56.6
  S42IL-141 86.3 78.9
  S42IL-143 85.1 85.4
  Scarlett  88.6 69.6

 treatment:genotype:year
, , year = 2018

         genotype
treatment Barke HOR10151 S42IL-141 S42IL-143 Scarlett
  control 89.4  86.4     85.3      85.6      91.0
  drought 56.5  57.2     87.4      84.6      86.1

, , year = 2019

         genotype
treatment Barke HOR10151 S42IL-141 S42IL-143 Scarlett
  control 87.5  84.6     84.2      84.7      82.4
  drought 27.1  28.6     73.6      86.2      56.9
*************************************************************************
***************************************************************
```

2. Length of the Main Spike

```
Shapiro-Wilk normality test

data:  resspikelength
W = 0.98852, p-value = 0.09021


summary(anovaspikelength)
                         Df Sum Sq Mean Sq F value   Pr(>F)
treatment                 1  234.7  234.69 275.384  < 2e-16 ***
genotype                  4  846.9  211.73 248.448  < 2e-16 ***
year                      1   23.0   22.96  26.946 5.35e-07 ***
treatment:genotype        4   15.0    3.75   4.399  0.00201 **
treatment:year            1    0.7    0.74   0.867  0.35286
genotype:year             4    2.9    0.74   0.863  0.48750
treatment:genotype:year   4    5.4    1.36   1.593  0.17782
Residuals               190  161.9    0.85
---
```

```
Signif. codes:  0 â€˜***â€™ 0.001 â€˜**â€™ 0.01 â€˜*â€™ 0.05 â€˜.â€™ 0.1
â€˜ â€™ 1
> print(model.tables(anovaspikelength,"means"),digits=3)
Tables of means
Grand mean

9.12381

 treatment
treatment
control drought
  10.18    8.07

 genotype
genotype
    Barke  HOR10151 S42IL-141 S42IL-143  Scarlett
     8.90      5.34     10.93     10.48      9.96

 year
year
2018 2019
9.65 8.91

 treatment:genotype
         genotype
treatment Barke HOR10151 S42IL-141 S42IL-143 Scarlett
  control 10.26  6.43    11.55     11.40     11.26
  drought  7.55  4.25    10.30      9.56      8.67

 treatment:year
         year
treatment 2018  2019
  control 10.61 10.01
  drought  8.68  7.82

 genotype:year
           year
genotype    2018  2019
  Barke      9.50  8.67
  HOR10151   5.55  5.26
  S42IL-141 11.70 10.62
  S42IL-143 11.07 10.25
  Scarlett  10.42  9.78

 treatment:genotype:year
, , year = 2018

         genotype
treatment Barke HOR10151 S42IL-141 S42IL-143 Scarlett
  control 10.75  6.22    12.67     11.75     11.67
  drought  8.25  4.88    10.73     10.38      9.17

, , year = 2019
```

```
         genotype
treatment Barke HOR10151 S42IL-141 S42IL-143 Scarlett
  control 10.07  6.51    11.10     11.27     11.10
  drought  7.27  4.00    10.13      9.23      8.47
*************************************************************************
***************************************************
```

3. Wilting Score

```
Shapiro-Wilk normality test

data:  resWS
W = 0.99198, p-value = 0.3041

  summary(anovaWS)
                         Df Sum Sq Mean Sq  F value   Pr(>F)
treatment                 1  477.9   477.9 2683.797  < 2e-16 ***
genotype                  4  122.9    30.7  172.549  < 2e-16 ***
year                      1    0.1     0.1    0.782  0.37767
treatment:genotype        4  114.0    28.5  160.014  < 2e-16 ***
treatment:year            1    8.8     8.8   49.388 3.66e-11 ***
genotype:year             4    2.3     0.6    3.171  0.01499 *
treatment:genotype:year   4    2.5     0.6    3.565  0.00788 **
Residuals               190   33.8     0.2
---
Signif. codes:  0 â€˜***â€™ 0.001 â€˜**â€™ 0.01 â€˜*â€™ 0.05 â€˜.â€™ 0.1
â€˜ â€™ 1
> print(model.tables(anovaWS,"means"),digits=3)
Tables of means
Grand mean

1.705714

 treatment
treatment
control drought
   0.20    3.21

 genotype
genotype
    Barke  HOR10151 S42IL-141 S42IL-143  Scarlett
    2.214     2.883     1.050     0.790     1.590

 year
year
 2018  2019
1.665 1.722

 treatment:genotype
         genotype
treatment Barke HOR10151 S42IL-141 S42IL-143 Scarlett
  control 0.24  0.22     0.20      0.13      0.20
  drought 4.19  5.55     1.90      1.45      2.98
```

```
 treatment:year
         year
treatment 2018 2019
  control 0.48 0.08
  drought 2.85 3.36

 genotype:year
           year
genotype    2018  2019
  Barke     1.917 2.333
  HOR10151  2.733 2.943
  S42IL-141 1.217 0.983
  S42IL-143 0.808 0.783
  Scarlett  1.650 1.567

 treatment:genotype:year
, , year = 2018

         genotype
treatment Barke HOR10151 S42IL-141 S42IL-143 Scarlett
  control 0.50  0.55     0.52      0.45      0.38
  drought 3.33  4.92     1.92      1.17      2.92

, , year = 2019

         genotype
treatment Barke HOR10151 S42IL-141 S42IL-143 Scarlett
  control 0.13  0.09     0.07      0.00      0.13
  drought 4.53  5.80     1.90      1.57      3.00
```

****
****

4. Grain weight/plant

```
Shapiro-Wilk normality test

data:  resGrain_DW
W = 0.98853, p-value = 0.09076


summary(anovaGrain_DW)
                         Df Sum Sq Mean Sq  F value  Pr(>F)
treatment                 1   4751    4751 2205.606 < 2e-16 ***
genotype                  4    415     104   48.179 < 2e-16 ***
year                      1      1       1    0.418 0.51891
treatment:genotype        4    266      67   30.885 < 2e-16 ***
treatment:year            1     15      15    6.979 0.00894 **
genotype:year             4     12       3    1.430 0.22563
treatment:genotype:year   4     18       5    2.120 0.07984 .
Residuals               190    409       2
---
Signif. codes:  0 â€˜***â€™ 0.001 â€˜**â€™ 0.01 â€˜*â€™ 0.05 â€˜.â€™ 0.1
â€˜ â€™ 1
```

```
> print(model.tables(anovaGrain_DW,"means"),digits=3)
Tables of means
Grand mean

8.135014

 treatment
treatment
control drought
  12.89    3.38

 genotype
genotype
    Barke  HOR10151 S42IL-141 S42IL-143  Scarlett
     7.32      6.00      8.99     10.11      8.26

 year
year
2018 2019
8.24 8.09

 treatment:genotype
         genotype
treatment Barke HOR10151 S42IL-141 S42IL-143 Scarlett
  control 13.91 10.40    12.95     13.53     13.67
  drought  0.73  1.59     5.03      6.69      2.85

 treatment:year
         year
treatment 2018  2019
  control 12.57 13.02
  drought  3.90  3.17

 genotype:year
           year
genotype    2018  2019
  Barke      7.54  7.23
  HOR10151   6.32  5.87
  S42IL-141  8.33  9.25
  S42IL-143 10.38 10.00
  Scarlett   8.62  8.12

 treatment:genotype:year
, , year = 2018

         genotype
treatment Barke HOR10151 S42IL-141 S42IL-143 Scarlett
  control 13.68  9.83    11.33     14.01     14.01
  drought  1.41  2.81     5.33      6.74      3.23

, , year = 2019

         genotype
treatment Barke HOR10151 S42IL-141 S42IL-143 Scarlett
```

```
  control 14.00 10.63    13.59     13.34     13.54
  drought  0.46  1.10     4.90      6.67      2.70
*************************************************************************
****************************************************

5. Plant height
Shapiro-Wilk normality test

data:  resplant_height
W = 0.99101, p-value = 0.2189
summary(anovaplant_height)
                         Df Sum Sq Mean Sq F value   Pr(>F)    
treatment                 1  27915   27915 892.913  < 2e-16 ***
genotype                  4    767     192   6.133 0.000116 ***
year                      1    640     640  20.476 1.06e-05 ***
treatment:genotype        4    239      60   1.909 0.110498    
treatment:year            1    769     769  24.605 1.56e-06 ***
genotype:year             4    124      31   0.989 0.414495    
treatment:genotype:year   4    301      75   2.407 0.050976 .  
Residuals               190   5940      31                     
---
Signif. codes:  0 â€˜***â€™ 0.001 â€˜**â€™ 0.01 â€˜*â€™ 0.05 â€˜.â€™ 0.1
â€˜ â€™ 1
> print(model.tables(anovaplant_height,"means"),digits=3)
Tables of means
Grand mean

81.6361

 treatment
treatment
control drought 
   93.2    70.1 

 genotype
genotype
    Barke  HOR10151 S42IL-141 S42IL-143  Scarlett 
     84.0      81.9      80.0      83.3      79.0 

 year
year
2018 2019 
84.4 80.5 

 treatment:genotype
         genotype
treatment Barke HOR10151 S42IL-141 S42IL-143 Scarlett
  control 94.7  94.9     90.1      94.6      91.5    
  drought 73.3  68.9     69.8      72.1      66.5    

 treatment:year
         year
treatment 2018 2019
  control 92.9 93.3
```

```
  drought 75.9 67.8

 genotype:year
           year
genotype    2018 2019
  Barke     84.6 83.8
  HOR10151  84.7 80.8
  S42IL-141 83.3 78.6
  S42IL-143 86.2 82.2
  Scarlett  83.3 77.3

 treatment:genotype:year
, , year = 2018

         genotype
treatment Barke HOR10151 S42IL-141 S42IL-143 Scarlett
  control 92.3  96.5     88.0      96.7      91.0
  drought 76.8  72.8     78.6      75.7      75.5

, , year = 2019

         genotype
treatment Barke HOR10151 S42IL-141 S42IL-143 Scarlett
  control 95.7  94.3     91.0      93.7      91.7
  drought 71.9  67.4     66.2      70.6      62.9
*************************************************************************
********************
6. Grain number per main spike
Shapiro-Wilk normality test

data:  resgrainnumber_mainspike
W = 0.98342, p-value = 0.51436

summary(anovagrainnumber_mainspike)
                         Df Sum Sq Mean Sq F value   Pr(>F)    
treatment                 1  11470   11470 432.871  < 2e-16 ***
genotype                  4   3316     829  31.282  < 2e-16 ***
year                      1    122     122   4.601   0.0332 *  
treatment:genotype        4   5208    1302  49.134  < 2e-16 ***
treatment:year            1   1186    1186  44.750 2.44e-10 ***
genotype:year             4    159      40   1.498   0.2045    
treatment:genotype:year   4   1073     268  10.125 1.89e-07 ***
Residuals               190   5035      26                     
---
Signif. codes:  0 â€˜***â€™ 0.001 â€˜**â€™ 0.01 â€˜*â€™ 0.05 â€˜.â€™ 0.1
â€˜ â€™ 1
> print(model.tables(anovagrainnumber_mainspike,"means"),digits=3)
Tables of means
Grand mean

20.42857

 treatment
treatment
```

```
control drought 
  27.82   13.04 

 genotype 
genotype
    Barke  HOR10151 S42IL-141 S42IL-143  Scarlett 
    13.10     23.24     23.79     22.64     19.38 

 year 
year
 2018  2019 
21.63 19.95 

 treatment:genotype 
         genotype
treatment Barke HOR10151 S42IL-141 S42IL-143 Scarlett
  control 22.7  38.7     25.0      26.0      26.7    
  drought  3.5   7.8     22.6      19.3      12.0    

 treatment:year 
         year
treatment 2018  2019 
  control 25.27 28.84
  drought 18.00 11.05

 genotype:year 
           year
genotype    2018  2019 
  Barke     13.42 12.97
  HOR10151  22.33 23.60
  S42IL-141 25.08 23.27
  S42IL-143 25.25 21.60
  Scarlett  22.08 18.30

 treatment:genotype:year 
, , year = 2018

         genotype
treatment Barke HOR10151 S42IL-141 S42IL-143 Scarlett
  control 20.5  27.3     26.5      26.3      25.7    
  drought  6.3  17.3     23.7      24.2      18.5    

, , year = 2019

         genotype
treatment Barke HOR10151 S42IL-141 S42IL-143 Scarlett
  control 23.6  43.2     24.4      25.9      27.1    
  drought  2.3   4.0     22.1      17.3       9.5    
*************************************************************************
**********************
```

7. Tiller number

Shapiro-Wilk normality test

```
data:  restiller_number
W = 0.98711, p-value = 0.05402

 summary(anovatiller_number)
                         Df Sum Sq Mean Sq  F value   Pr(>F)    
treatment                 1   3497    3497 1200.472  < 2e-16 ***
genotype                  4    195      49   16.732 9.13e-12 ***
year                      1     19      19    6.408  0.01217 *  
treatment:genotype        4    104      26    8.894 1.33e-06 ***
treatment:year            1    450     450  154.427  < 2e-16 ***
genotype:year             4     56      14    4.809  0.00102 ** 
treatment:genotype:year   4     74      19    6.354 8.07e-05 ***
Residuals               190    554       3                      
---
Signif. codes:  0 â€˜***â€™ 0.001 â€˜**â€™ 0.01 â€˜*â€™ 0.05 â€˜.â€™ 0.1
â€˜ â€™ 1
> print(model.tables(anovatiller_number,"means"),digits=3)
Tables of means
Grand mean
         
16.20476 

 treatment
treatment
control drought 
  20.29   12.12 

 genotype
genotype
    Barke  HOR10151 S42IL-141 S42IL-143  Scarlett 
    16.14     14.88     16.64     17.74     15.62 

 year
year
 2018  2019 
15.73 16.39 

 treatment:genotype
         genotype
treatment Barke HOR10151 S42IL-141 S42IL-143 Scarlett
  control 20.95 19.48    20.05     20.81     20.14   
  drought 11.33 10.29    13.24     14.67     11.10   

 treatment:year
         year
treatment 2018  2019 
  control 17.50 21.40
  drought 13.97 11.39

 genotype:year
           year
genotype    2018  2019 
  Barke     16.67 15.93
  HOR10151  15.08 14.80
```

```
  S42IL-141 15.92 16.93
  S42IL-143 15.92 18.47
  Scarlett  15.08 15.83

 treatment:genotype:year
, , year = 2018

         genotype
treatment Barke HOR10151 S42IL-141 S42IL-143 Scarlett
  control 18.83 16.17    17.17     18.33     17.00
  drought 14.50 14.00    14.67     13.50     13.17

, , year = 2019

         genotype
treatment Barke HOR10151 S42IL-141 S42IL-143 Scarlett
  control 21.80 20.80    21.20     21.80     21.40
  drought 10.07  8.80    12.67     15.13     10.27
*************************************************************************
******************************************************
```

8. Fresh weight

```
Shapiro-Wilk normality test

data:  resfreshwght
W = 0.98965, p-value = 0.7246

summary(anovafreshwght)
                         Df Sum Sq Mean Sq F value   Pr(>F)
treatment                 1  11970   11970 118.466  < 2e-16 ***
genotype                  4  17116    4279  42.350  < 2e-16 ***
year                      1  26198   26198 259.282  < 2e-16 ***
treatment:genotype        4   8053    2013  19.925 1.03e-13 ***
treatment:year            1    104     104   1.032  0.31094
genotype:year             4   3460     865   8.561 2.26e-06 ***
treatment:genotype:year   4   1482     371   3.667  0.00667 **
Residuals               190  19197     101
---
Signif. codes:  0 â€˜***â€™ 0.001 â€˜**â€™ 0.01 â€˜*â€™ 0.05 â€˜.â€™ 0.1
â€˜ â€™ 1
> print(model.tables(anovafreshwght,"means"),digits=3)
Tables of means
Grand mean

34.91013

 treatment
treatment
control drought
   42.5    27.4

 genotype
genotype
    Barke  HOR10151 S42IL-141 S42IL-143  Scarlett
```

```
     48.5      21.5      39.5      30.3      34.8

 year
year
2018 2019
52.6 27.8

 treatment:genotype
         genotype
treatment Barke HOR10151 S42IL-141 S42IL-143 Scarlett
  control 64.8  25.0     53.3      31.7      37.5
  drought 32.3  17.9     25.7      28.9      32.0

 treatment:year
         year
treatment 2018 2019
  control 59.0 35.8
  drought 46.1 19.9

 genotype:year
           year
genotype    2018 2019
  Barke     69.6 40.1
  HOR10151  29.3 18.3
  S42IL-141 52.4 34.3
  S42IL-143 51.0 22.0
  Scarlett  60.6 24.5

 treatment:genotype:year
, , year = 2018

         genotype
treatment Barke HOR10151 S42IL-141 S42IL-143 Scarlett
  control 80.1  28.6     62.8      56.8      66.8
  drought 59.1  30.0     42.0      45.2      54.3

, , year = 2019

         genotype
treatment Barke HOR10151 S42IL-141 S42IL-143 Scarlett
  control 58.7  23.6     49.5      21.7      25.8
  drought 21.5  13.1     19.2      22.4      23.1

*************************************************************************
***************************************************

9. SPike Number
Shapiro-Wilk normality test

data:  resspikenumber
W = 0.98547, p-value = 0.5103

summary(anovaspikenumber)
                         Df Sum Sq Mean Sq  F value   Pr(>F)
```

```
treatment                1   4781    4781 2133.861  < 2e-16 ***
genotype                 4    146      37   16.296 1.71e-11 ***
year                     1   2228    2228  994.360  < 2e-16 ***
treatment:genotype       4    154      39   17.187 4.76e-12 ***
treatment:year           1     29      29   12.967 0.000405 ***
genotype:year            4    136      34   15.154 9.03e-11 ***
treatment:genotype:year  4     29       7    3.254 0.013084 *
Residuals              190    426       2
---
Signif. codes:  0 â€˜***â€™ 0.001 â€˜**â€™ 0.01 â€˜*â€™ 0.05 â€˜.â€™ 0.1
â€˜ â€™ 1
> print(model.tables(anovaspikenumber,"means"),digits=3)
Tables of means
Grand mean

19.33333

 treatment
treatment
control drought
  24.10   14.56

 genotype
genotype
    Barke  HOR10151 S42IL-141 S42IL-143  Scarlett
    18.86     18.64     19.38     20.93     18.86

 year
year
 2018  2019
14.18 21.39

 treatment:genotype
         genotype
treatment Barke HOR10151 S42IL-141 S42IL-143 Scarlett
  control 24.52 24.24    23.52     24.38     23.86
  drought 13.19 13.05    15.24     17.48     13.86

 treatment:year
         year
treatment 2018  2019
  control 18.37 26.40
  drought 10.00 16.39

 genotype:year
           year
genotype    2018  2019
  Barke     13.67 20.93
  HOR10151  15.75 19.80
  S42IL-141 13.00 21.93
  S42IL-143 14.58 23.47
  Scarlett  13.92 20.83

 treatment:genotype:year
```

```
, , year = 2018

         genotype
treatment Barke HOR10151 S42IL-141 S42IL-143 Scarlett
  control 18.83 20.33    16.83     18.33     17.50
  drought  8.50 11.17     9.17     10.83     10.33

, , year = 2019

         genotype
treatment Barke HOR10151 S42IL-141 S42IL-143 Scarlett
  control 26.80 25.80    26.20     26.80     26.40
  drought 15.07 13.80    17.67     20.13     15.27

************************************************************************
*********************************************************
Gas exchange, 15 days after stress
************************************************************************
*********************************************************
10. Transpirtion rate (E)

Shapiro-Wilk normality test

data:  resE
W = 0.98116, p-value = 0.7441

summary(anovaE)
                         Df    Sum Sq   Mean Sq F value   Pr(>F)    
treatment                 1 0.0003816 0.0003816 1892.22  < 2e-16 ***
genotype                  4 0.0000348 0.0000087   43.09  < 2e-16 ***
year                      1 0.0004534 0.0004534 2248.09  < 2e-16 ***
treatment:genotype        4 0.0000319 0.0000080   39.51  < 2e-16 ***
treatment:year            1 0.0001519 0.0001519  753.33  < 2e-16 ***
genotype:year             4 0.0000139 0.0000035   17.23 4.48e-12 ***
treatment:genotype:year   4 0.0000127 0.0000032   15.74 3.85e-11 ***
Residuals               190 0.0000383 0.0000002                     
---
Signif. codes:  0 â€˜***â€™ 0.001 â€˜**â€™ 0.01 â€˜*â€™ 0.05 â€˜.â€™ 0.1
â€˜ â€™ 1
> print(model.tables(anovaE,"means"),digits=3)
Tables of means
Grand mean

0.002329967

 treatment
treatment
control drought
0.00368 0.00098

 genotype
genotype
    Barke  HOR10151 S42IL-141 S42IL-143  Scarlett
 0.001974  0.001956  0.003039  0.002510  0.002170
```

```
 year
year
   2018    2019
0.00001 0.00326

 treatment:genotype
         genotype
treatment Barke   HOR10151 S42IL-141 S42IL-143 Scarlett
  control 0.00351 0.00354  0.00470   0.00310   0.00354
  drought 0.00044 0.00037  0.00138   0.00192   0.00080

 treatment:year
         year
treatment 2018    2019
  control 0.00001 0.00515
  drought 0.00000 0.00137

 genotype:year
           year
genotype    2018    2019
  Barke     0.00001 0.00276
  HOR10151  0.00001 0.00274
  S42IL-141 0.00001 0.00425
  S42IL-143 0.00001 0.00351
  Scarlett  0.00001 0.00303

 treatment:genotype:year
, , year = 2018

         genotype
treatment Barke   HOR10151 S42IL-141 S42IL-143 Scarlett
  control 0.00001 0.00001  0.00001   0.00001   0.00001
  drought 0.00000 0.00000  0.00000   0.00001   0.00000

, , year = 2019

         genotype
treatment Barke   HOR10151 S42IL-141 S42IL-143 Scarlett
  control 0.00491 0.00496  0.00658   0.00434   0.00495
  drought 0.00062 0.00052  0.00193   0.00268   0.00112
*************************************************************************
*************************************************************************
*****************
```

11. Net Co2 Assimilation rate (A)

```
Shapiro-Wilk normality test

data:  resA
W = 0.97315, p-value = 0.7177

summary(anovaA)
                         Df Sum Sq Mean Sq F value   Pr(>F)
treatment                 1  10794   10794 5160.57  < 2e-16 ***
```

```
genotype                 4    813     203   97.13  < 2e-16 ***
year                     1   2341    2341 1118.99  < 2e-16 ***
treatment:genotype       4    797     199   95.23  < 2e-16 ***
treatment:year           1     39      39   18.62 2.57e-05 ***
genotype:year            4    185      46   22.15 5.05e-15 ***
treatment:genotype:year  4     54      13    6.44 7.01e-05 ***
Residuals              190    397       2
---
Signif. codes:  0 â€˜***â€™ 0.001 â€˜**â€™ 0.01 â€˜*â€™ 0.05 â€˜.â€™ 0.1
â€˜ â€™ 1
> print(model.tables(anovaA,"means"),digits=3)
Tables of means
Grand mean

15.69796

 treatment
treatment
control drought
  22.87    8.53

 genotype
genotype
    Barke  HOR10151 S42IL-141 S42IL-143  Scarlett
    15.33     13.63     16.83     18.88     13.82

 year
year
 2018  2019
20.98 13.59

 treatment:genotype
         genotype
treatment Barke HOR10151 S42IL-141 S42IL-143 Scarlett
  control 24.82 22.34    22.68     23.01     21.49
  drought  5.85  4.92    10.97     14.75      6.15

 treatment:year
         year
treatment 2018  2019
  control 28.83 20.48
  drought 13.13  6.69

 genotype:year
           year
genotype    2018  2019
  Barke     22.47 12.48
  HOR10151  18.69 11.61
  S42IL-141 19.70 15.68
  S42IL-143 25.40 16.27
  Scarlett  18.62 11.89

 treatment:genotype:year
, , year = 2018
```

```
         genotype
treatment Barke HOR10151 S42IL-141 S42IL-143 Scarlett
  control 32.3  27.8     27.6      30.5      26.0
  drought 12.7   9.6     11.8      20.3      11.3

, , year = 2019

         genotype
treatment Barke HOR10151 S42IL-141 S42IL-143 Scarlett
  control 21.8  20.2     20.7      20.0      19.7
  drought  3.1   3.1     10.6      12.5       4.1
*************************************************************************
*************************************************************************
************************
```

12.Intracellular Co2 (Ci)

```
Shapiro-Wilk normality test

data:  resCi
W = 0.98927, p-value = 0.81943

summary(anovaCi)
                         Df Sum Sq Mean Sq F value   Pr(>F)    
treatment                 1  75826   75826  374.96  < 2e-16 ***
genotype                  4  20391    5098   25.21  < 2e-16 ***
year                      1   3694    3694   18.27 3.03e-05 ***
treatment:genotype        4  32764    8191   40.50  < 2e-16 ***
treatment:year            1  45051   45051  222.77  < 2e-16 ***
genotype:year             4  24991    6248   30.89  < 2e-16 ***
treatment:genotype:year   4  26930    6733   33.29  < 2e-16 ***
Residuals               190  38423     202                     
---
Signif. codes:  0 â€˜***â€™ 0.001 â€˜**â€™ 0.01 â€˜*â€™ 0.05 â€˜.â€™ 0.1
â€˜ â€™ 1
> print(model.tables(anovaCi,"means"),digits=3)
Tables of means
Grand mean

225.3296

 treatment
treatment
control drought
  244.3   206.3

 genotype
genotype
    Barke  HOR10151 S42IL-141 S42IL-143  Scarlett
    221.1     217.9     238.0     213.6     236.0

 year
year
```

```
 2018  2019 
218.7 228.0 

 treatment:genotype 
         genotype
treatment Barke HOR10151 S42IL-141 S42IL-143 Scarlett
  control 242.0 244.6    275.2     217.3     242.7   
  drought 200.2 191.2    200.9     210.0     229.4   

 treatment:year 
         year
treatment 2018  2019 
  control 260.9 237.7
  drought 176.5 218.2

 genotype:year 
           year
genotype    2018  2019 
  Barke     214.9 223.6
  HOR10151  232.0 212.2
  S42IL-141 229.8 241.3
  S42IL-143 217.9 211.9
  Scarlett  198.8 250.9

 treatment:genotype:year 
, , year = 2018

         genotype
treatment Barke HOR10151 S42IL-141 S42IL-143 Scarlett
  control 253.2 278.6    266.1     246.5     259.9   
  drought 176.6 185.5    193.6     189.3     137.7   

, , year = 2019

         genotype
treatment Barke HOR10151 S42IL-141 S42IL-143 Scarlett
  control 237.5 231.0    278.8     205.6     235.8   
  drought 209.7 193.5    203.8     218.2     266.0   
*************************************************************************
*************************************************************************
********

13.Stomatal Conductance (gsw)
Shapiro-Wilk normality test

data:  resgsw
W = 0.98666, p-value = 0.06565

summary(anovagsw)
                         Df Sum Sq Mean Sq  F value   Pr(>F)    
treatment                 1 2.6433  2.6433 2602.609  < 2e-16 ***
genotype                  4 0.1293  0.0323   31.821  < 2e-16 ***
year                      1 0.5011  0.5011  493.387  < 2e-16 ***
treatment:genotype        4 0.2125  0.0531   52.310  < 2e-16 ***
```

```
treatment:year            1 0.1705  0.1705  167.865  < 2e-16 ***
genotype:year             4 0.0666  0.0166   16.387  1.5e-11 ***
treatment:genotype:year   4 0.0243  0.0061    5.982 0.000148 ***
Residuals               190 0.1930  0.0010
---
Signif. codes:  0 â€˜***â€™ 0.001 â€˜**â€™ 0.01 â€˜*â€™ 0.05 â€˜.â€™ 0.1
â€˜ â€™ 1
> print(model.tables(anovagsw,"means"),digits=3)
Tables of means
Grand mean

0.1922355

 treatment
treatment
control drought
 0.3044  0.0800

 genotype
genotype
    Barke  HOR10151 S42IL-141 S42IL-143  Scarlett
   0.1867    0.1701    0.2373    0.1972    0.1699

 year
year
  2018   2019
0.2695 0.1613

 treatment:genotype
         genotype
treatment Barke HOR10151 S42IL-141 S42IL-143 Scarlett
  control 0.323 0.300    0.376     0.251     0.272
  drought 0.050 0.040    0.099     0.143     0.068

 treatment:year
         year
treatment 2018  2019
  control 0.427 0.256
  drought 0.112 0.067

 genotype:year
           year
genotype    2018   2019
  Barke     0.2781 0.1501
  HOR10151  0.2798 0.1262
  S42IL-141 0.2643 0.2265
  S42IL-143 0.2856 0.1619
  Scarlett  0.2395 0.1421

 treatment:genotype:year
, , year = 2018

         genotype
treatment Barke HOR10151 S42IL-141 S42IL-143 Scarlett
```

```
  control 0.451 0.482    0.426     0.395     0.380
  drought 0.105 0.078    0.103     0.177     0.099

, , year = 2019

         genotype
treatment Barke HOR10151 S42IL-141 S42IL-143 Scarlett
  control 0.271 0.227    0.356     0.194     0.229
  drought 0.029 0.025    0.097     0.130     0.055
*************************************************************************
*************************************************************************
******************
```

14. Vapour pressure deficit of the leaf (VPDleaf)

```
Shapiro-Wilk normality test

data:  resVPDleaf
W = 0.97926, p-value = 0.5789

summary(anovaVPDleaf)
                         Df Sum Sq Mean Sq  F value   Pr(>F)    
treatment                 1  2.361   2.361  296.297  < 2e-16 ***
genotype                  4  0.331   0.083   10.399 1.23e-07 ***
year                      1 20.048  20.048 2516.302  < 2e-16 ***
treatment:genotype        4  1.380   0.345   43.293  < 2e-16 ***
treatment:year            1  8.627   8.627 1082.828  < 2e-16 ***
genotype:year             4  2.450   0.613   76.890  < 2e-16 ***
treatment:genotype:year   4  0.277   0.069    8.684 1.85e-06 ***
Residuals               190  1.514   0.008                      
---
Signif. codes:  0 â€˜***â€™ 0.001 â€˜**â€™ 0.01 â€˜*â€™ 0.05 â€˜.â€™ 0.1
â€˜ â€™ 1
> print(model.tables(anovaVPDleaf,"means"),digits=3)
Tables of means
Grand mean

2.273983

 treatment
treatment
control drought
  2.168   2.380

 genotype
genotype
    Barke  HOR10151 S42IL-141 S42IL-143  Scarlett
    2.279     2.226     2.231     2.315     2.319

 year
year
 2018  2019
2.763 2.079

 treatment:genotype
```

```
         genotype
treatment Barke HOR10151 S42IL-141 S42IL-143 Scarlett
  control 2.051 2.223    2.063     2.252     2.251
  drought 2.508 2.228    2.399     2.378     2.386

 treatment:year
         year
treatment 2018 2019
  control 2.34 2.10
  drought 3.19 2.06

 genotype:year
           year
genotype    2018  2019
  Barke     2.967 2.004
  HOR10151  2.487 2.121
  S42IL-141 2.883 1.970
  S42IL-143 2.639 2.185
  Scarlett  2.836 2.112

 treatment:genotype:year
, , year = 2018

         genotype
treatment Barke HOR10151 S42IL-141 S42IL-143 Scarlett
  control 2.48  2.23     2.37      2.21      2.38
  drought 3.45  2.74     3.40      3.06      3.29

, , year = 2019

         genotype
treatment Barke HOR10151 S42IL-141 S42IL-143 Scarlett
  control 1.88  2.22     1.94      2.27      2.20
  drought 2.13  2.02     2.00      2.10      2.02
*************************************************************************
*************************************************************************
**********************************
```

15. Electron transport rate (ETR)

Shapiro-Wilk normality test

```
data:  resETR
W = 0.99375, p-value = 0.6416

summary(anovaETR)
                         Df Sum Sq Mean Sq F value   Pr(>F)    
treatment                 1  15474   15474   79.17 4.48e-16 ***
genotype                  4  72095   18024   92.21  < 2e-16 ***
year                      1  17597   17597   90.03  < 2e-16 ***
treatment:genotype        4  10735    2684   13.73 7.45e-10 ***
treatment:year            1  45914   45914  234.91  < 2e-16 ***
genotype:year             4  14212    3553   18.18 1.17e-12 ***
treatment:genotype:year   4  16105    4026   20.60 4.08e-14 ***
```

```
Residuals               190  37137     195
---
Signif. codes:  0 â€˜***â€™ 0.001 â€˜**â€™ 0.01 â€˜*â€™ 0.05 â€˜.â€™ 0.1
â€˜ â€™ 1
> print(model.tables(anovaETR,"means"),digits=3)
Tables of means
Grand mean

116.2573

 treatment
treatment
control drought
  124.8   107.7

 genotype
genotype
    Barke  HOR10151 S42IL-141 S42IL-143  Scarlett
     99.4     100.5     114.4     150.6     116.4

 year
year
 2018  2019
130.7 110.5

 treatment:genotype
         genotype
treatment Barke HOR10151 S42IL-141 S42IL-143 Scarlett
  control 107.8 121.2    119.8     149.4     126.0
  drought  90.9  79.7    109.0     151.8     106.9

 treatment:year
         year
treatment 2018  2019
  control 162.7 109.7
  drought  98.8 111.2

 genotype:year
           year
genotype    2018  2019
  Barke     138.8  83.6
  HOR10151  114.8  94.8
  S42IL-141 118.7 112.7
  S42IL-143 160.2 146.7
  Scarlett  121.2 114.6

 treatment:genotype:year
, , year = 2018

         genotype
treatment Barke HOR10151 S42IL-141 S42IL-143 Scarlett
  control 190.3 154.5    147.6     189.3     131.7
  drought  87.3  75.0     89.9     131.1     110.7
```

```
, , year = 2019

         genotype
treatment Barke HOR10151 S42IL-141 S42IL-143 Scarlett
  control  74.8 107.9    108.7     133.4     123.7
  drought  92.4  81.6    116.7     160.1     105.4
*************************************************************************
*************************************************************************
***********************
```

16. Intrinsic water use efficiency (iWUE)

```
Shapiro-Wilk normality test

data:  resiWUE
W = 0.98835, p-value = 0.06955

summary(anovaiWUE)
                         Df Sum Sq Mean Sq F value   Pr(>F)    
treatment                 1  41755   41755 404.786  < 2e-16 ***
genotype                  4  10817    2704  26.215  < 2e-16 ***
year                      1     34      34   0.333    0.564    
treatment:genotype        4  17540    4385  42.508  < 2e-16 ***
treatment:year            1  10108   10108  97.987  < 2e-16 ***
genotype:year             4   3953     988   9.581 4.45e-07 ***
treatment:genotype:year   4   4714    1179  11.425 2.49e-08 ***
Residuals               190  19599     103                     
---
Signif. codes:  0 â€˜***â€™ 0.001 â€˜**â€™ 0.01 â€˜*â€™ 0.05 â€˜.â€™ 0.1
â€˜ â€™ 1
> print(model.tables(anovaiWUE,"means"),digits=3)
Tables of means
Grand mean

94.01245

 treatment
treatment
control drought
   79.9   108.1

 genotype
genotype
    Barke  HOR10151 S42IL-141 S42IL-143  Scarlett
     97.0     101.0      86.5     101.1      84.4

 year
year
2018 2019
93.4 94.3

 treatment:genotype
         genotype
treatment Barke HOR10151 S42IL-141 S42IL-143 Scarlett
```

```
  control  80.2  80.0     60.8      97.2      81.4
  drought 113.9 122.0    112.3     105.1      87.3

 treatment:year
         year
treatment 2018  2019 
  control  68.3  84.6
  drought 118.4 104.0

 genotype:year
           year
genotype    2018  2019 
  Barke      96.4  97.3
  HOR10151   90.5 105.2
  S42IL-141  89.8  85.2
  S42IL-143  96.3 103.1
  Scarlett   93.9  80.5

 treatment:genotype:year
, , year = 2018

         genotype
treatment Barke HOR10151 S42IL-141 S42IL-143 Scarlett
  control  71.4  57.8     65.2      77.5      69.6
  drought 121.4 123.1    114.3     115.1     118.3

, , year = 2019

         genotype
treatment Barke HOR10151 S42IL-141 S42IL-143 Scarlett
  control  83.7  88.9     59.0     105.0      86.1
  drought 110.9 121.5    111.5     101.1      74.9
```
